# The effects of limb position and grasped load on hand gesture classification using electromyography, force myography, and their combination

**DOI:** 10.1371/journal.pone.0321319

**Published:** 2025-04-10

**Authors:** Peyton R. Young, Kihun Hong, Eden J. Winslow, Giancarlo K. Sagastume, Marcus A. Battraw, Richard S. Whittle, Jonathon S. Schofield

**Affiliations:** 1 Department of Mechanical and Aerospace Engineering, University of California—Davis, Davis, California, United States of America; 2 Department of Biomedical Engineering, University of California—Davis, Davis, California, United States of America; 3 Department of Electrical and Computer Engineering, University of California—Davis, Davis, California, United States of America; 4 Department of Mechanical and Mechatronic Engineering and Advanced Manufacturing, California State University, Chico, Chico, California, United States of America; Polytechnic University of Marche: Universita Politecnica delle Marche, ITALY

## Abstract

Hand gesture classification is crucial for the control of many modern technologies, ranging from virtual and augmented reality systems to assistive mechatronic devices. A prominent control technique employs surface electromyography (EMG) and pattern recognition algorithms to identify specific patterns in muscle electrical activity and translate these to device commands. While being well established in consumer, clinical, and research applications, this technique suffers from misclassification errors caused by limb movements and the weight of manipulated objects, both vital aspects of how we use our hands in daily life. An emerging alternative control technique is force myography (FMG) which uses pattern recognition algorithms to predict hand gestures from the axial forces present at the skin’s surface created by contractions of the underlying muscles. As EMG and FMG capture different physiological signals associated with muscle contraction, we hypothesized that each may offer unique additional information for gesture classification, potentially improving classification accuracy in the presence of limb position and object loading effects. Thus, we tested the effect of limb position and grasped load on 3 different sensing modalities: EMG, FMG, and the fused combination of the two. 27 able-bodied participants performed a grasp and release task with 4 hand gestures at 8 positions and under 5 object weight conditions. We then examined the effects of limb position and grasped load on gesture classification accuracy across each sensing modality. It was found that position and grasped load had statistically significant effects on the classification performance of the 3 sensing modalities and that the combination of EMG and FMG provided the highest classification accuracy of hand gesture, limb position, and grasped load combinations (97.34%) followed by FMG (92.27%) and then EMG (82.84%). This points to the fact that the addition of FMG to traditional EMG control systems offers unique additional data for more effective device control and can help accommodate different limb positions and grasped object loads.

## Introduction

From manipulating objects and tools to communication, our hands are a vital part of our daily interactions with the world and others around us. As many consumers and clinical technologies continue to advance, our hand gestures are becoming more utilized as a mode of control making non-invasive gesture recognition systems increasingly important with such devices. Commercial systems and current research use hand positioning and movements for a variety of different interfaces, spanning from virtual reality to aiding in sign-language communication [1–4]. One field that relies heavily on hand gestures is the field of assistive and rehabilitative mechatronics. Many devices such as upper limb prostheses or hand exoskeletons depend on gesture classification for effective control [5–7]. Thus, a major thrust in the research of such assistive devices is to optimize control techniques that decode hand gestures to achieve effective and reliable device control.

Depending on the application, there are many ways to decode hand gestures spanning from optical techniques to wearable sensors that can differentiate between varying gestures [1–4,8,9]. One of the most commonly used approaches pairs surface electromyography (EMG) with machine learning algorithms for gesture recognition [10]. This technique places multiple EMG sensors on the skin’s surface to measure electrical muscle activity resulting from activation of the muscles associated with hand movements. Electrodes are most usually positioned on the forearm although positioning over hand intrinsic muscle may also be used [11,12]. In patient populations where the hand itself is missing yet the forearm musculature remains (ex. transradial or wrist disarticulation amputations) EMG and machine learning algorithms can be used to distinguish patterns of muscle activity and identify the user’s intended missing hand movements, which is then translated into device actuation [13].

Even though widely employed across a variety of applications, EMG control systems exhibit limitations. Pattern recognition algorithms require a degree of consistency in the incoming EMG signals to achieve reliable device control. In practical applications, especially for individuals using devices such as assistive hand exoskeletons or prostheses, the users will often require the device to assist them as they manipulate and interact with objects. This can cause EMG signal variance as muscles within the forearm contract to grasp an object and stabilize the wrist, hand, and/or limb in the required position. All the resulting muscle activity can then be directly influenced by the weight (load) of the object itself. Variances in the output of the EMG sensors can negatively impact the classification accuracy as the system’s control algorithms are often trained by the user in a single unloaded position, leading to incorrect classification of hand gestures and unreliable device control [14–17]. The degradation of pattern classification specifically related to limb movement is an issue that has been termed the position effect [18,19], a prominent challenge for EMG control systems. For prosthesis users, position and load effects can cause the EMG sensors embedded in the prosthetic socket to lift away from the surface of the skin in some areas while pressing more firmly into the skin in other areas. This variability in EMG contact changes impedance values often resulting in inconsistent gesture classification [16,18].

One potential approach to mitigate position and load effects is by implementing a multi-modal combination of sensors. The additional unique and complementary information provided by various sensing modalities can more completely capture the muscle activity resulting from different hand gestures, limb positions, and grasped loads; potentially providing a more robust technique for classifying gestures. One potential complementary sensing modality to EMG is a technique known as force myography (FMG). First described in the 1960s [20], FMG measures normal forces at the skin’s surface generated during muscle contractions and the corresponding local changes in muscle volume or shape [21], often captured using multiple force or pressure sensors (typically force sensitive resistors, or FSRs). Like EMG, FMG-based control can use machine learning algorithms to differentiate between patterns of muscle activity by capturing pressure changes across the sensors during hand gestures. FMG has been implemented in a variety of experiments, demonstrating its ability to classify hand gestures, finger, elbow, and shoulder movements [21–25]. Much of the appeal of FMG systems stems from the fact that the driving circuitry is often less complex when compared to EMG systems, requiring less componentry, facilitating their ease of fabrication and cost effectiveness [25]. Further, FMG has been found to provide classification accuracies that are similar to, and sometimes better than, that of EMG [21,26]. Additional advantages of FMG include that the sensors are typically not electrically sensitive to skin sweat and cross talk [27–29], challenges that must be overcome for effective implementation of EMG systems. While FMG systems do suffer from potential hysteresis and drift errors [30–32], FMG provides an additional bio-signal when paired with EMG as it records changes in muscle volume or shape rather than underlaying muscles’ electrical activity [27]. Thus, while both sensing modalities have limitations, both record unique information about muscle activity that offer the potential to be combined for more robust classification of hand gestures, mitigating challenges associated with limb position and object load effects.

In fact, in recent works, the efficacy of the combination of EMG and FMG has begun to be explored. Ke et al. designed and fabricated an EMG+FMG sensor package, and tested its ability to classify hand gestures, demonstrating that that the combined EMG and FMG sensor yielded gesture classification accuracies upwards of 97% [33]. Ahmadizadeh et al. used FMG sensors paired with commercially purchased EMG sensors to classify multiple hand gestures during dynamic movements in a transradial amputee subject and found that the addition of EMG to their FMG system added no statistical improvements to classification accuracy in each of their testing conditions as compared to FMG alone [34]. A study by Chen et al. created an experimental EMG+FMG system to classify 22 hand gestures using a variety of different classification models. They found that the addition of FMG to EMG added additional robustness to their system and yielded higher classification accuracies [35]. A similar test was conducted by Jiang et al. who tested an experimental EMG+FMG band for hand gesture recognition for able-bodied participants and found that the addition of FMG increased classification accuracy by about 10% when compared to EMG [36]. Finally, a study from Nowak et al. used an EMG+FMG system to classify hand gestures using a variety of different signal features and sensor configurations and found that FMG performed statistically better than EMG during hand gesture classification and yielded less error while the combination of EMG and FMG was found to not improve classification accuracy when compared to solely FMG [37].

While these studies have begun to demonstrate the potential of combining EMG and FMG for hand gesture classification, there is no consensus on whether their combination statistically improves classification performance [33–37]. Additionally, most previous research either tested and compared these sensing modalities individually or, if combined, did so at a single limb position and/or without considering the load effects of grasped objects. For example, while there have been multiple studies that examined how limb position affects classification accuracy for EMG systems [18,38–41], this body of literature often neglects the interaction with objects (load effects). Therefore, such approaches may differ significantly from how we interact within the real world, moving our limbs to different positions and interacting with objects of varying weights.

Our work expands upon the current body of research by quantifying hand gesture performance of EMG, FMG, and their combination (EMG+FMG) under conditions more representative of the real world that incorporate changes in limb position and manipulated object loads. We hypothesized that the combination of EMG and FMG would provide higher gesture classification accuracies across a variety of different positions and grasped loads when compared to EMG and FMG separately. However, we further anticipated that variations in both position and grasped weight would still lead to a decrease in classification accuracy across all sensing modalities.

## Methods

We recruited N=27 able-bodied participants between the ages of 18–35 years old (17 male, 10 female, average age = 23.7, standard deviation (SD) = 3.04). Research protocols were approved by the Institutional Review Board at the University of California, Davis and participants provided written, informed consent. Recruitment began on July 28^th^, 2023 and the last participant was recruited on April 17^th^, 2024. N=27 was strategically selected after performing an a priori power analysis with an effect size of 0.5, alpha value of 0.05, and power of 0.8 on data collected from 21 participants by our EMG, FMG and EMG+FMG systems [42]. Prior to the experiment, participants completed a demographic and handedness survey [43], as well as had measurements taken of their forearm circumference and length. Participants performed the experiment with their dominant hand.

### EMG+FMG band

**Fig 1:**
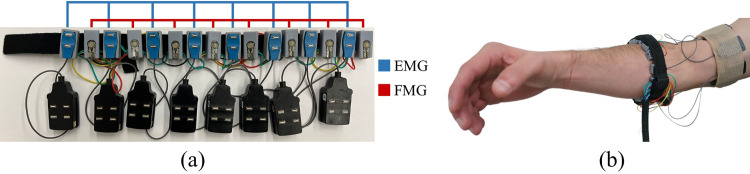
The EMG+FMG band used for the experiment. We employed 8 EMG sensors and 8 FMG sensors which were housed in 3D printed housings and placed in an alternating sequence on a Velcro strap. The sensors were positioned such that they could be spaced equidistantly around the participant’s forearm before the band was strapped to the arm.

To collect both EMG and FMG data, we designed and fabricated a custom EMG+FMG armband that housed both sensor types and was donned on each participant’s dominant forearm (Fig 1).

Our armband incorporated 8 Trigno Mini EMG Sensors (Delsys Inc., Boston, USA). 8 sensors were selected for size considerations and to meet the minimum number of EMG sensors suggested to avoid negative impact on classification performance [44]. For our FMG system, we used 8 Interlink Electronics FSR400 FSR sensors to ensure consistency with prior literature [24,45–49]. While we acknowledged that FSRs have known limitations including hysteresis and drift error, they were selected as they are commonly implemented through literature and offer advantages related to ease of use, implementation, and low cost [25,30–32]. The FSRs were placed in 3D printed housings and spaced equidistantly on a Velcro strap in an alternating sequence, allowing adjustments to accommodate varying forearm sizes. While we chose to place these sensors in this manner, it has been suggested that this along with a variety of other sensor configurations provide no significant difference in gesture classification accuracy [37]. The recordings from both the EMG and FMG sensing modalities were captured using 2 National Instrument USB 6210 Data Acquisition Systems, one for each sensing modality, at a rate of 2000 Hz and saved for offline analysis using a custom MATLAB code.

### Experimental design

**Fig 2:**
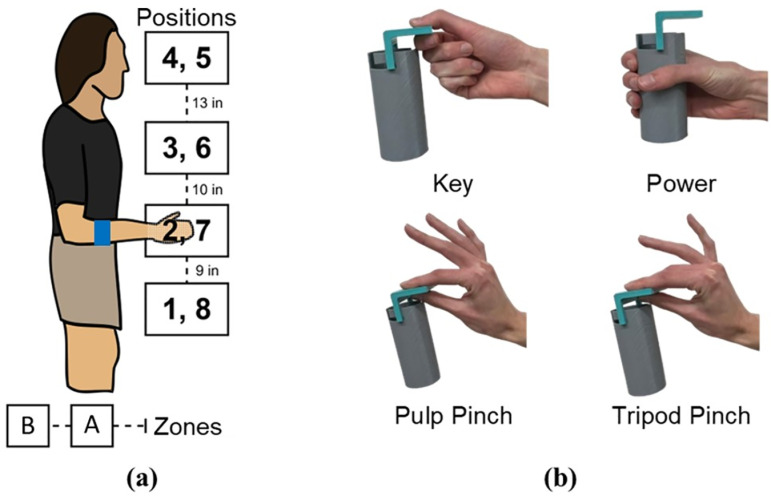
The reaching positions, standing zones, and hand gestures. (a) The reaching positions and standing zones for the experiment [42]. The subject would reach to positions 1-4 while standing at zone A and positions 5-8 while standing at zone B. The participant’s elbow was bent at 90 degrees at position 2, between 90 degrees and fully extended for positions 1, 3, and 4, and fully extended for positions 5-8. (b) The 4 hand gestures used for the experiment along with the 3D printed manipulandum. The manipulandum was made of two parts which allowed it to be top loaded and grasped with a variety of hand gestures. The weights used for the experiment consisted of a no weight condition (the weight of the manipulandum, 53 g), 250g, 500g, 750g, and 1000g.

Participants stood in front of a shelving unit (described further below) and were instructed to grasp objects (manipulanda) of varying weights at varying positions (in line with the sagittal plane of their dominant arm) using prompted hand grasp configurations (Fig 2a and 2b); collectively manipulating hand grasp, object weight, and limb position. While this experiment followed a similar paradigm to Radmand et al., it differed as it included physical object manipulations rather than performing hand grasps in space without grasping objects [19,21].

As shown in Fig 2b, we 3D-printed a manipulandum designed to be loaded with different weights and held using 4 different hand grasps: Key, Pulp Pinch, Power, and Tripod Pinch. These represent 4 of the 7 most frequently used hand grasps in activities of daily living [50]. The weights used for the experiment were 0 grams added (the weight of the manipulandum, 53 grams), 250 grams, 500 grams, 750 grams, and 1000 grams. These values were based on work by Feix et al. in which they found the typical weight of manipulated household objects to be on average 500 grams and range from nearly weightless to 1000 grams [50].

Participants stood in front of a shelving unit at zone A (the close distance) or zone B (the far distance), to reach and grasp in 8 positions, as shown in Fig 1a. Participants stood at zone A for reaching positions 1–4 to simulate the “close” positions and stood at zone B for reaching positions 5–8 to simulate the “far” positions. The placements of both the reaching positions and standing zones were determined by participant height and thus varied between participants to ensure that the participant could comfortably grasp the object at all positions [21]. The manipulandum was placed in line with the sagittal plane of the participant’s dominant arm and could be moved either forward or backward on the shelf to ensure the participant could grasp it comfortably at the specific position.

### Testing protocol

#### Setup.

The experimental set up began with first aligning the reaching positions and standing zones for each participant. The participant would face the shelving system and bend their dominant arm to 90 degrees, as measured by a goniometer. A shelf was moved to this position and marked as position 2 and used to set up the remaining shelving heights, as illustrated by Fig 2a. The standing zones were then marked as positions that allowed the participant’s forearm to be fully extended at positions 5–8 and between 90 degrees and fully extended for positions 1–4 [21,42].

After the positions and zones were set, participants would then don the EMG+FMG band which was worn around the muscle bulk of the forearm at a position approximately 2/3 of the distance from the distal end [51]. The EMG and FMG channels were then tested by having the participant perform 3 voluntary maximum contractions. During these contractions we visually inspected the data and adjusted the armband’s positioning to ensure that the sensors were recording muscle activity data while minimizing noise.

#### Testing.

A custom MATLAB script was used to both collect data as well as queue the participants to interact with the manipulandum. During testing, participants stood at the appropriate zone, raised their arm to the first prompted position, and relaxed their limb. The MATLAB script queued participants via an auditory tone to grasp the manipulandum with one of the 4 hand grasps and lift it, holding it for 3 seconds before releasing and relaxing for 3 seconds. This process was repeated a total of 3 times before the manipulandum was moved to a different position. After the participant completed all 8 positions, the test was repeated for the remaining weights and then for all remaining hand grasps. The order of the hand grasps, weights, and positions was randomized to ensure that any potential muscle fatigue did not influence EMG or FMG recordings on a specific hand grasp, weight, or position combination. In total, each participant performed 480 grasps, which took approximately 3 hours with multiple opportunities for rest to mitigate the effects of fatigue.

### Data processing

#### Data segmentation.

Using event time stamps collected by the MATLAB script, we segmented our data into “contraction” and “rest” phases which isolated data from when the manipulandum was grasped and when the participant was not grasping the manipulandum, respectively. Using these time stamps, we were able to select the time that was directly in the middle of the 3 second contraction. This point was then used to isolate 70% of the data (35% from either side of the middle point) to avoid capturing muscle states in-between contraction and relaxation periods and to ensure consistency across contractions. This resulted in 2.1 seconds of data per contraction and 6.3 seconds of data for each hand grasp, weight, and position combination. The “contraction” data for each hand grasp, weight, and position was parsed together and segmented using a 200ms window and a 50ms time increment, as has been suggested in literature [52].

#### Feature extraction.

Next, features were extracted from the sensor data from each of the segmented windows to create feature vectors for pattern classification [17]. As we used 3 sensing modality types (EMG, FMG, and EMG+FMG), we used 3 separate feature extraction techniques. For EMG feature extraction, we used the Hudgins Set, which contains the following features: mean absolute values, slope sign changes, waveform length, zero crossings, and root mean squared [53,54]. For FMG feature extraction we used a single feature, the mean absolute value of the data, which has been shown to provide effective gesture classification performance in prior literature [21]. For EMG+FMG these same features were extracted and then combined to create an EMG+FMG feature vector, a combination technique known as “stacked” [37]. Although it is possible that more advanced feature sets and combination techniques may be more robust to position and loading effects, these features were selected for EMG and FMG as we wanted to investigate how position and loading effects alter the classification accuracy of the sensing modalities in their most basic form. That is, we aimed to characterize performance using features that are among the most widely reported in literature; thus, helping this work establish a performance baseline that may serve as a point of comparison for other more advanced feature sets and machine learning approaches.

#### Pattern classification.

The feature vectors were then used to train a linear discriminant analysis (LDA) classifier which was selected for its simplicity of implementation, minimal computational demands, and ease of training [17]. While there are other pattern recognition models such as Support Vector Machines or Random Forest Classification that may have the potential to be more robust to limb position and loading effects, LDA was chosen as we wanted to establish a performance baseline using a classifier that is among the most commonly reported in literature [55–58]. To find classification accuracy, we implemented leave-one-out cross validation to train and test our classifier [59,60]. This technique trained the classifier on all but one of the hand gesture repetitions in the data set. The trained classifier was then tested with the “left out” repetition and the classification accuracy was recorded. This process was repeated with a different repetition left out until all combinations of training and testing were achieved. The predictions made by the classifier were then compared to the correct values and used to create confusion matrices to tabulate the accuracy of each sensing modality. This was done for varying combinations of position and grasped loads described in the statistical analysis section below. The overall accuracy of each confusion matrix, or the classification accuracy, was calculated by averaging the diagonal elements of the confusion matrix which correspond to the percentage of correct predictions.

### Statistical analysis

**Fig 3:**
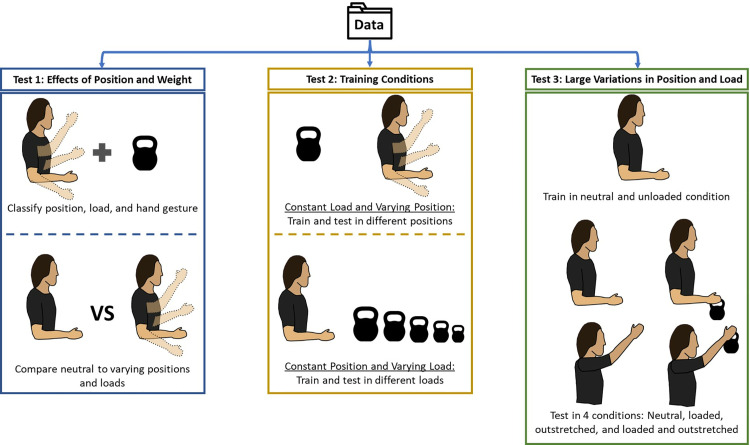
Overview of the 3 tests conducted in the experiment. Test 1 was split into 2 analyses, as shown by the dashed line. The first analysis examined how accurately each of the sensing modalities could classify combinations of limb position, grasped load, and hand gesture. The second analysis compared classifying hand gestures in the neutral and unloaded position to varying limb positions and grasped loads. Test 2 was also split into 2 analyses and investigated different training and testing conditions, the first being a constant grasped load with varying limb positions and the second being a constant limb position with varying grasped loads. Test 3 characterized how each sensing modality was affected by large variations in limb position and grasped load by training the model in a neutral and unloaded condition and testing it in 4 other conditions.

To examine statistical differences between the classification performance of each sensing modality and to test for significant effects of position and grasped load, we used multiple linear mixed effect (LME) models, with subject as a random intercept to account for the repeated measures design. The LME models were fit using restricted maximum likelihood. Diagnostic plots for all models were examined visually to confirm normality and homoscedasticity of residuals. For all models, an alpha value of 0.05 was used to determine significance between data sets. The analyses were grouped into 3 tests which are shown pictorially in Fig 3 and described and detailed in the following subsections. For each test, classification accuracies were aggregated by sensing modality across participants for statistical comparisons.

#### Test 1: The effects of position and grasped load.

This test was comprised of 2 analyses that were used to test how the position and grasped load affect each sensing modality and draw comparisons between them.

***Variations in position, grasped load, and hand grasp:*** The first analysis investigated how accurately each sensing modality could predict changes in combinations of grasp pattern, grasped load, and position, providing information as to which sensing modality performed the best under variations in these 3 variables. For each participant, 3 confusion matrixes were created (1 for each sensing modality) encompassing every combination of grasp, grasped load, and position resulting in a 160x160 element matrix. We then calculated the classification accuracy of these confusion matrices by averaging the diagonal elements to quantify how well each sensing modality could correctly predict a specific grasp, weight, and position pattern. The classification accuracies for each sensing modality were aggregated across all participants and then averaged to find the average classification accuracy for EMG, FMG, and their combination. To create an overarching confusion matrix for each sensing modality, we averaged the confusion matrices across all participants (shown in the results section).

We then used a LME model to examine if the sensing modalities were significantly different from one another. In the model, classification accuracy was the dependent variable, the sensing modality type was used for the fixed effect, and the participant ID was the random effect. To calculate differences between sensing modality, we calculated the estimated marginal means (EMM) and then used pairwise comparisons using the Benjamini and Hochberg false discovery rate (FDR) correction to adjust and decrease the chances of type 1 error [61].

***Neutral vs. varying:*** The next analyses compared the classification accuracy of the 4 hand gestures at a neutral and unloaded position to the classification accuracy of the hand gestures when the positions and weights were varied. This allowed us to quantify how varying the positions and grasped loads could affect overall gesture classification. The neutral and unloaded position was defined as the participant grasping the unloaded manipulandum with their elbow bent at 90 degrees (position 2). This position was chosen as it is commonly used for training hand gesture recognition systems [17]. We again employed leave-one-out cross validation, using feature vectors from each of the four hand gestures to create a 4x4 matrix with the hand gestures being the true and predicted labels. The neutral condition contained features from only the neutral and unloaded position while the varying conditions contained features from all position and weight combinations. From these confusion matrices, the classification accuracies for each sensing modality were calculated and then compared using a LME which used the accuracy as the dependent variable, sensing modality as the fixed effect, and participant ID as the random effect.

#### Test 2: Training conditions.

We then wanted to investigate the effect of training the classifier in a single position or weight and testing in the others. This would allow us to investigate the effect that position and grasped load has on each sensing modality when trained at single position and weight, as is a common technique employed in literature [48,54,62]. In previous work [42] we investigated how training the sensing modalities in the conventional unloaded, neutral position, then testing in an outstretched and loaded condition yields much lower and more variable classification accuracies for each sensing modality, pointing to the fact that varying the position and weight influenced the classification accuracies. To expand on this concept, we created 2 separate conditions: Constant weight with varying positions and constant position with varying weights. These conditions helped isolate the effects of limb position and grasped load by keeping one constant and varying the other. For the constant weight and varying position test, we began by collecting all the data from a certain weight (i.e., 0, 250, 500, 750, and 1000g). For each weight, we would then start at position 1 and train the classifier at that position to classify the 4 hand gestures. The classifier was then tested with data from the remaining positions and the overall accuracy was recorded. This process was repeated for each of the remaining positions and then repeated for each weight condition. Data from all participants at each constant load was aggregated together by sensing modality to portray the spread of classification accuracies over our sample population. For the constant position test, a similar testing method was used except the position was kept constant and the classifier was trained and tested on the varying weights.

After these accuracies were calculated for each position and weight combination, we used multiple LME models to examine their effects. For the constant weight and varying position condition, we employed 5 LME models, one for each weight. Each model used accuracy as the dependent variable, sensing modality accuracy as the fixed effect, and participant ID as the random effect. Just as done before, the EMM were calculated for each sensing modality and pairwise comparisons using an FDR adjustment were used to draw comparisons across the sensing modalities. For the constant position and varying weight condition, 8 LME models (one for each position) were used and each of them used the same dependent variable and fixed and random effects as the previous condition.

#### Test 3: Large variations in position and grasped load.

We then tested the extent that each sensing modality was affected by large variations in limb position and grasped load. This was done using a similar scheme to the previous test where the classifier was trained at the neutral and unloaded position (position 2, 0 grams) to classify the 4 hand gestures (pinch, power, key, and tripod). The classifier was then tested at the 4 most extreme conditions: At the neutral and unloaded position (baseline test, termed “neutral”), at the most outstretched position without load (position 5, 0 grams, termed “outstretched”), at the neutral position but heavily loaded (position 2, 1000 grams, termed “loaded”), and at the most outstretched and loaded position (position 5, 1000 grams, termed “outstretched and loaded”). The classification accuracies from all 4 conditions were calculated and averaged across all participants by sensing modality. The results were then compared in 2 ways using LME models: 1) The same sensing modality across each testing condition and 2) across sensing modalities within the testing condition. The positions and grasped loads chosen for this test represent the most variation from the neutral position to demonstrate how large changes in both affect each sensing modality when trained at the neutral position.

## Results

### The effects of position and grasped load

#### Classifying hand gesture, grasped load, and position combinations.

**Fig 4:**
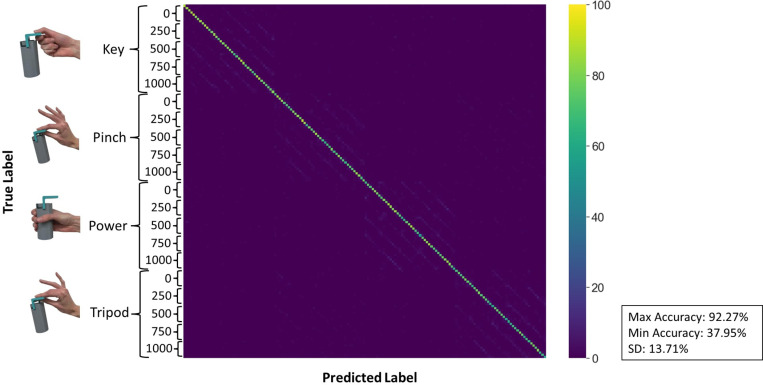
The average confusion matrix for classifying combinations of hand gesture, grasped load, and limb position for FMG. The confusion matrix is broken down into 4 sections, one for each hand grasp (key, pinch, power, and tripod). These sections are further broken down into the 5 grasped loads (0, 250, 500, 750, and 1000g) which are subsequently broken down into 8 positions, ordered 1-8. The color bar on the side visually shows the percent of times a label is classified as another. Darker colors indicate lower percent while lighter colors correspond to a higher percent. The maximum and minimum average accuracies across the participants are included along with the standard deviation of the average classification accuracies.

We first investigated the impact of variation of position and grasped load on the accuracy of each sensing modality when classifying different combinations of grasp, grasped load, and limb position. This resulted in a 160x160 confusion matrix for each sensing modality per participant (3 matrices per participant). We averaged these confusion matrices for each sensing modality to find maximum and minimum classification accuracies and the combinations they corresponded with. The average confusion matrix for FMG is depicted in Fig 4 and is also pictured in the supplementary material along with the other sensing modality’s average confusion matrices (S1–S3 Figs).

From Fig 4, S2, and S3 Figs, we found which hand grasp, grasped load, and limb position combination yielded the maximum and minimum classification accuracies on average across participants. The combination that resulted in the minimum accuracy for FMG was tripod with 250 grams at position 7 (54.95 ±32.57%) while the maximum accuracy was found at key with 500 grams at position 2 (86.71±15.18%). For EMG, the minimum accuracy was at the combination of tripod with 500 grams at position 8 (52.12±18.64%) while the maximum accuracy occurred at key with 0 grams at position 2 (90.27±9.79%). Finally, for EMG+FMG, power with 750 grams at position 8 produced the minimum classification accuracy (79.43±20.47%) while key with 0 grams at position 2 corresponded to the maximum accuracy (98.43±3.35%).

**Fig 5:**
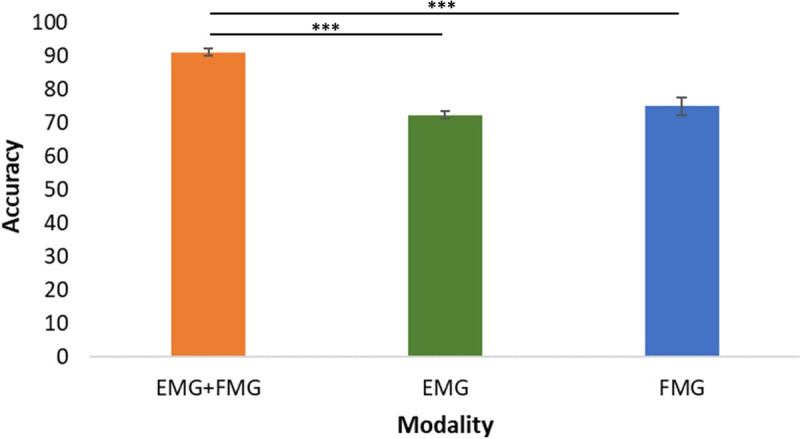
Average classification accuracies for each sensing modality classifying hand grasp, grasped load, and limb position combinations. These accuracies were calculated by averaging the classification accuracies for each participant per sensing modality. Using a LME model, statistical differences were found between EMG+FMG and the two sensing modalities separately (p<0.001). Further, EMG and FMG were found to be not statistically different from one another.

Using these confusion matrices, the classification accuracy for each sensing modality was found by taking the average across the diagonal for each of these matrices for each participant. Accuracy values for each sensing modality were aggregated and then averaged across all 27 participants to create an average classification accuracy for each. A bar graph displaying these average values is shown in Fig 5.

As shown, the combination of EMG and FMG produced higher prediction accuracies than either of them by themselves, yielding an average accuracy of 91% and a standard error of 0.99%. Further, the classification accuracies of EMG and FMG were found to not be statistically different from one another and yielded standard errors of 1.14% and 2.59%, respectively. To further illustrate variance in the data, the standard deviation for each sensing modality was calculated and it was found that FMG (13.71%) yielded the largest variance followed by EMG (5.92%) and then EMG+FMG (5.25%). The numerical values for the classification accuracies, standard errors, and standard deviations are displayed in Table 1.

**Table 1:** The average classification accuracy (%), standard error, and standard deviation for each sensing modality for test 1.

Sensing Modality	Accuracy (%)	Standard Error (%)	Standard Deviation (%)
**EMG+FMG**	91.02	1.01	5.25
**EMG**	72.23	1.14	5.92
**FMG**	74.89	2.64	13.71

#### Comparing the neutral position to varying limb positions and grasped loads.

**Fig 6:**
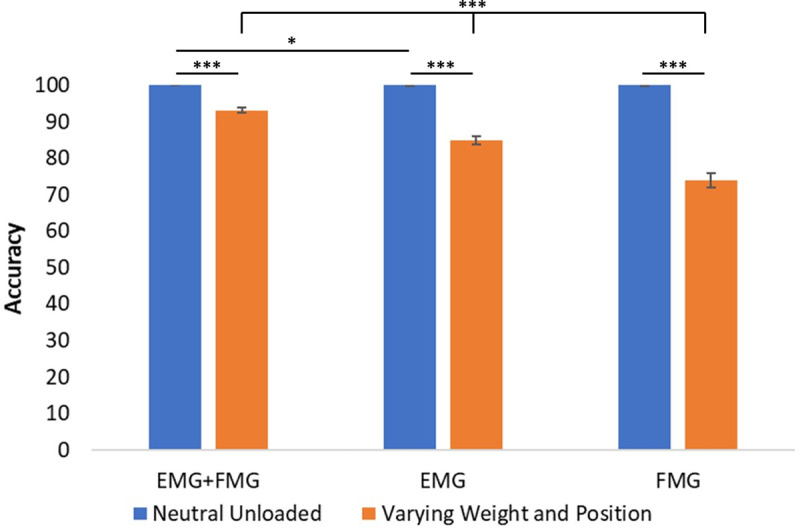
The average classification accuracy for each sensing modality at the neutral and unloaded position compared to varying grasped loads and limb positions. Averages were calculated by aggregating and then averaging all participant’s classification accuracies by sensing modality. For the neutral and unloaded position, EMG was found to be statistically different from EMG+FMG. For the varying weight and position condition, all sensing modalities were found to be statistically different from one another. Further, when comparing within a sensing modality, it was found that the neutral and unloaded condition was statistically different than the varying weight and position condition. * p < 0.05, ** p < 0.01, *** p < 0.001.

We then examined the difference between classifying the 4 hand gestures at the neutral and unloaded position to classifying them under varying grasped loads and positions. This is shown in Fig 6 which statistically compares the averaged classification accuracies at the unloaded and neutral position to the results when grasped loads and limb positions were varied.

As shown by Fig 6, no statistical differences were found between EMG+FMG and FMG when at the neutral and unloaded position, with the classification accuracy of the 4 hand gestures averaging close to 100% and exhibiting standard errors close to 0. Interestingly, our model illustrated a statistical difference between EMG+FMG and EMG at the neutral position. For the varied grasped loads and position condition, each sensing modality was found to be statistically different from one another, with EMG+FMG performing the best with an average classification accuracy of 93% followed by EMG (85%) and then FMG (74%). Further, when comparing across the 2 conditions for the same sensing modality, it was found that the average classification accuracies were statistically different. These classification accuracies along with the standard error and deviation are displayed numerically in Table 2.

**Table 2:** The average accuracy, standard error, and standard deviation for comparing the classification accuracy of the 4 hand gestures at the neutral and unloaded condition to the varied weight and position condition.

Condition	Sensing Modality	Accuracy (%)	Standard Error (%)	Standard Deviation (%)
**Neutral and Unloaded**	EMG+FMG	100.00	0.00	0.00
	EMG	99.83	0.08	0.41
	FMG	99.97	0.03	0.16
**Varying Weight and Position**	EMG+FMG	93.14	0.67	3.47
	EMG	84.18	1.00	5.19
	FMG	73.84	1.96	10.17

As tabulated in Table 2, for the neutral and unloaded condition, standard error and standard deviation all remained close to 0, with EMG having the largest standard error and deviation (0.08%, 0.41%). However, for the varied weight and position condition, a larger variance was observed for all sensing modalities. FMG was found to yield the largest standard error and standard deviation (1.96%, 10.17%) followed by EMG (1%, 5.19%) and then EMG+FMG (0.67%, 3.47%), demonstrating that FMG had the largest amount of variance when classifying the 4 hand gestures during varied positions and weights.

### Training effects

#### Constant grasped load and varying positions.

**Fig 7:**
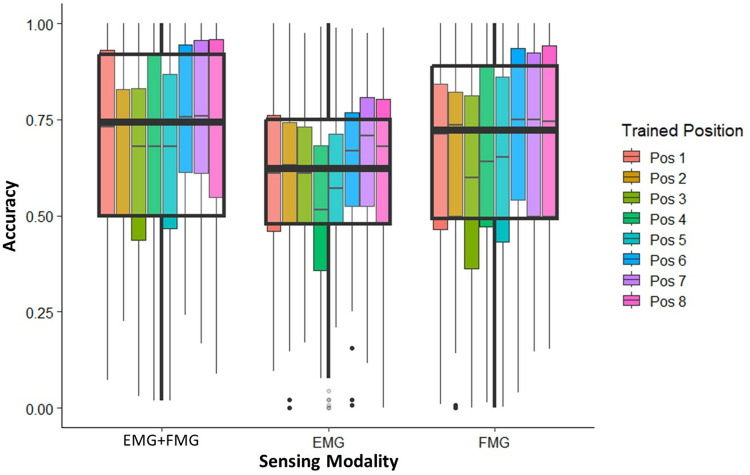
The spread of all participant’s gesture classification accuracy for EMG +FMG, EMG and FMG when trained at a single position and tested at the remaining positions under a constant grasped load of 500g. For each modality, the classifier was trained in one of 8 positions, as shown by the different colors in the legend. Once trained, it was tested in the remaining 7 positions. This was done at a constant grasped load of 500g. The overall variation of classification accuracy for each sensing modality is depicted by the black overlay which also shows the median classification accuracy.

We then examined the performance of each sensing modality when trained in a single position or weight and tested in others. To isolate the effects of position and grasped load on each sensing modality, we employed 2 cases: Constant grasped load with varying positions and constant limb position with varying grasped loads. The data for this analysis was found to be not normally distributed using a Shaprio-Wilk test and we thus reported our values as median and interquartile ranges to make comparisons. An example graph depicting the spread of classification accuracies for each modality under a constant grasped load of 500g is shown in Fig 7.

Fig 7 depicts the average accuracies of the 4 hand gestures when trained at a specific limb position and tested in another (represented by the different colors in the legend) while subjected to a constant load of 500 grams. This figure along with the remaining graphs for the constant load condition can be found in S4–S8 Figs. As shown, each sensing modality demonstrated large amounts of variability when trained at one position and tested at the remaining positions. As shown in Fig 7, based on the median value, EMG+FMG demonstrated numerically larger classification values (roughly 75%) followed by FMG and then EMG.

The median values for each sensing modality for all constant weight conditions are highlighted in Table 3.

**Table 3:** The median of the classification accuracies from the constant weight and varied position tests reported with the interquartile range.

Grasped Load	Sensing Modality	Median Accuracy (%)	Interquartile Range
0g	EMG+FMG *	74.19	43.34
	EMG (*, ⸕)	52.03	34.20
	FMG (⸕)	73.37	43.70
250g	EMG+FMG (*, ⸸)	75.00	44.51
	EMG (*, ⸕)	59.04	30.49
	FMG (⸸, ⸕)	74.39	42.73
500g	EMG+FMG (*, ⸸)	74.19	41.92
	EMG (*, ⸕)	62.20	27.29
	FMG (⸸, ⸕)	72.15	39.68
750g	EMG+FMG (*, ⸸)	74.59	42.28
	EMG (*, ⸕)	64.84	28.66
	FMG (⸸, ⸕)	71.14	40.45
1000g	EMG+FMG (*, ⸸)	73.37	40.45
	EMG (*)	67.07	28.25
	FMG (⸸)	68.29	35.57

For each grasped load, sensing modalities that share the same letter are statistically different than each other (p < 0.05). The * is used when comparing EMG+FMG to EMG, the ⸸ is used when comparing EMG+FMG to FMG, and the ⸕ is used when comparing EMG to FMG.

Our results revealed statistical differences for all EMG+FMG weight conditions. For all cases except 1000 grams, EMG and FMG by themselves were found to be statistically different from one another. Further, large amounts of variance were observed, as accuracy values ranged from close to 100% to almost 0% for all sensing modalities. This variance is illustrated by the IQR values (ranging from 28–44%) which demonstrate where most of the data was centralized around the median. As shown by Table 3, training in a single position and testing in another yielded a large variance in classification accuracies and a decrease in overall performance.

#### Constant position and varying grasped loads.

**Fig 8:**
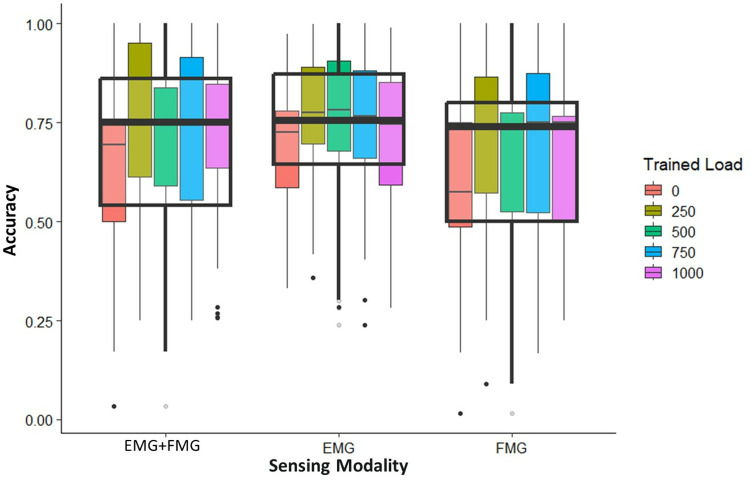
The spread of all participant’s gesture classification accuracy for EMG+FMG, EMG and FMG when trained at a single grasped load and tested at the remaining loads while at a constant position of position 1. For each modality, the classifier was trained at 1 of 5 loading conditions, as shown by the different colors in the legend. Once trained, the classifier was then tested in the remaining 4 loading conditions. This was done at a constant position of position 1. The overall variation of classification accuracy for each sensing modality is depicted by the black overlay which also shows the median classification accuracy.

We then performed the inverse test and kept the position constant while changing the grasped load to depict the effects caused by variations in grasped load. Fig 8 depicts sample data of the classification accuracies for all participants for the constant position of position 1.

Just as before, each exhibited large amounts of variance for each load they were trained in. As shown, on average, each of the sensing modalities achieved a median classification accuracy of roughly 75% when trained in a single weight. Fig 8 and the remaining graphs for the constant position condition can be found in S9–S16 Figs. Median values for each sensing modality for all constant positions are tabulated in Table 4.

**Table 4:** The median of the classification accuracies from the constant position and varied grasped loads tests reported with the interquartile range and the standard deviation.

Position	Sensing Modality	Median Accuracy (%)	Interquartile Range
1	EMG+FMG (*, ⸸)	75.00	31.96
	EMG (*, ⸕)	75.41	22.76
	FMG (⸸, ⸕)	73.98	30.08
2	EMG+FMG (*, ⸸)	73.48	28.91
	EMG (*, ⸕)	73.78	26.52
	FMG (⸸, ⸕)	70.73	26.88
3	EMG+FMG (⸸)	74.09	31.86
	EMG (⸕)	70.53	30.13
	FMG (⸸, ⸕)	69.51	26.83
4	EMG+FMG (*, ⸸)	73.07	32.98
	EMG (*)	66.67	26.83
	FMG (⸸)	68.09	26.22
5	EMG+FMG (*, ⸸)	75.00	35.42
	EMG (*, ⸕)	67.28	26.83
	FMG (⸸, ⸕)	75.000	38.26
6	EMG+FMG (⸸)	74.49	32.11
	EMG (⸕)	73.98	23.22
	FMG (⸸, ⸕)	71.04	32.11
7	EMG+FMG (*, ⸸)	72.97	35.06
	EMG (*, ⸕)	73.37	23.02
	FMG (⸸, ⸕)	67.99	30.28
8	EMG+FMG (*, ⸸)	75.00	35.87
	EMG (*, ⸕)	76.32	24.03
	FMG (⸸, ⸕)	70.63	32.98

For each position, sensing modalities that share the same letter are statistically different from each other (P < 0.05). The * is used when comparing EMG+FMG to EMG, the ⸸ is used when comparing EMG+FMG to FMG, and the ⸕ is used when comparing EMG to FMG.

As shown, there were statistical differences found between EMG+FMG and the other sensing modalities for all positions except for position 3 and position 6. At these positions, values from EMG+FMG were only statistically different from FMG. These results point to the fact that the combination of the two yields statistically different and numerically larger classification accuracies for most positions. It is good to note that each sensing modality demonstrated large amounts of variance, spanning from almost 0% to almost 100%. This is illustrated by the IQR values (ranging from 22–35%), depicting where the bulk of the average accuracies were found. For all positions, classification accuracies for EMG and FMG were found to not exhibit statistical differences from one another, hinting at the fact that these sensing modalities behave similarly to one another. As shown by Table 4, variations in weight effect the classification accuracy of each sensing modality and lead to a decrease in accuracy and large amounts of variance, a similar result to the one found from Table 3.

### Large variations in limb position and grasped load

**Fig 9:**
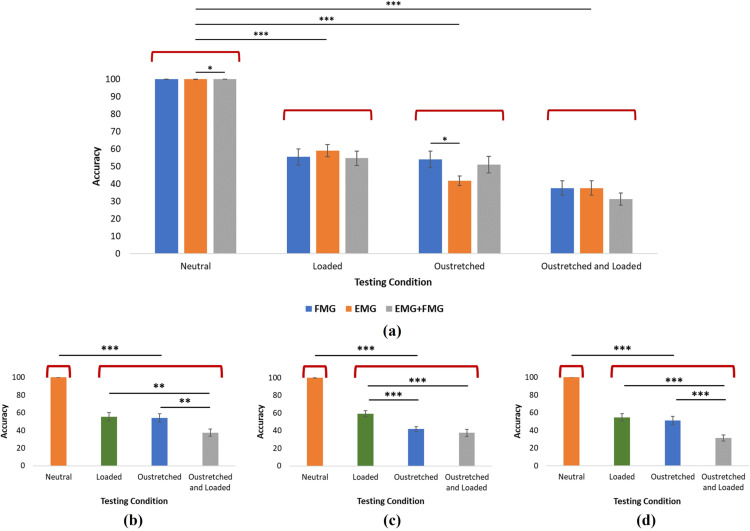
Average classification accuracies for each modality when training in the neutral and unloaded condition (position 2, 0g) and testing in the 4 most extreme conditions for each sensing modality: Neutral (position 2, 0g), Loaded (position 2, 1000g), Outstretched (position 5, 0g), and Outstretched and Loaded (position 5, 1000g). For each sensing modality, participant data was averaged to portray average classification accuracies over all participants. Fig 9a depicts how the neutral condition was statistically different from the other 3 conditions for each sensing modality (P < 0.001). Across sensing modalities for each condition, it was found that EMG and EMG+FMG were statistically different at the neutral condition and FMG and EMG were statistically different at the outstretched condition (P < 0.05). Fig 9b–9d compare across conditions for the same sensing modality (from right to left: FMG, EMG, EMG+FMG). It was found for all sensing modalities that the neutral condition was statistically different from all other conditions (P < 0.001). For the other conditions, it was found that loaded – outstretched, loaded – outstretched and loaded, and outstretched – outstretched and loaded were significantly different for FMG and EMG+FMG. For EMG, the loaded condition was significantly different from the others. * p < 0.05, ** p < 0.01, *** p < 0.001.

We further investigated how large variations in position and weight affected the gesture classification accuracy of each sensing modality. We did this by training the classifier in the neutral and unloaded position (position 2, 0 grams), then tested the classification accuracy under the most extreme conditions (4 conditions in total): At a neutral condition (position 2, 0 grams, termed “neutral”), at a maximum loaded condition (position 2, 1000 grams, termed “loaded”), at an outstretched condition (position 5, 0 grams, termed “outstretched”), and at an outstretched and loaded condition (position 5, 1000 grams, termed “outstretched and loaded”). The results of this are shown in Fig 9.

As shown in Fig 9a–9d, the addition of load and variation in position greatly affects classification accuracy of each of the sensing modalities. For all sensing modalities, the neutral condition was significantly different from each of the other testing conditions (P < 0.001) and yielded accuracies close to 100% and exhibiting little variance (as shown in Fig 6 as well). The other testing conditions however demonstrated more variance. For the loaded condition, no significant differences were found across sensing modalities, each yielding classification accuracies of roughly 55% with EMG reporting the highest value of 58%. This was a similar result to the outstretched and loaded condition where no significant differences were found across sensing modalities. At this condition, EMG and FMG both reported classification accuracies of roughly 37% while EMG+FMG yielded 31%. However, for the outstretched condition, it was found that FMG and EMG were significantly different from one another, yielding classification accuracies of 54% and 41% respectively. For this case, EMG+FMG was not significantly different from either of the sensing modalities and yielded an average classification accuracy of 51%.

We then compared the same sensing modalities across each of the testing conditions (i.e., FMG loaded compared to FMG outstretched). For FMG, there were no significant differences between the loaded and outstretched conditions and classification accuracies for both were found to be 55% and 54% respectively. However, the outstretched and loaded condition was found to be significantly different from the other 2 conditions and resulted in a classification accuracy of 37%. This trend was found to be the same for EMG+FMG which yielded classification accuracies of 54%, 51%, and 31% for the loaded, outstretched, and outstretched and loaded conditions (respectively). However, for EMG, it was found that the loaded condition was significantly different from the other 2 conditions when comparing classification accuracies. These results are tabulated and can be seen in Table 5.

**Table 5:** The average accuracy, standard error, and standard deviation for each of the sensing modalities when trained at the neutral condition and tested in the conditions listed.

Condition	Sensing Modality	Accuracy (%)	Standard Error (%)	Standard Deviation (%)
**Neutral**	EMG+FMG	100.00	0.00	0.00
	EMG	99.83	0.08	0.41
	FMG	99.97	0.03	0.16
**Loaded**	EMG+FMG	54.60	4.10	21.31
	EMG	58.91	3.45	17.92
	FMG	55.33	4.63	24.07
**Outstretched**	EMG+FMG	51.01	4.79	24.88
	EMG	41.72	2.77	14.38
	FMG	54.03	4.75	24.67
**Loaded and Outstretched**	EMG+FMG	31.30	18.19	3.50
	EMG	37.56	21.35	4.11
	FMG	37.51	21.39	4.12

## Discussion

The objective of this study was to explore how variations in limb position and grasped load affect the classification accuracy of 3 sensing modalities during hand gesture recognition: EMG, FMG, and the combination (EMG+FMG). In an offline analysis, it was found that when using LDA paired with leave-one-out cross validation that EMG+FMG was able to account for variations in position and grasped load better than EMG and FMG by themselves. This was illustrated by statistically different and higher classification accuracies when classifying hand gestures and combinations of hand gestures, grasped loads, and positions in space. However, when trained in a single position or weight condition and tested in another, it was found that all 3 sensing modalities yielded highly variable classification accuracies, demonstrating that the addition of another sensing modality does not yield better classifications if it is trained in a single position and weight condition.

### Additional information

It was found that EMG+FMG was more accurate in classifying combinations of hand grasp, grasped load, and limb position than the sensing modalities separately and yielded lower standard deviation and standard error values, demonstrating a more consistent classification accuracy. These results point to the fact that EMG and FMG provide unique additional information to each other. The combination of the two sensing modalities was found to create a richer set of data to aid in classifying the hand gesture along with where the limb is in space and how much load the limb was experiencing. This idea of unique additional information is further reinforced by the fact that EMG and FMG were found to be not statistically different from one another when classifying hand gesture, weight, and position, and yielded a lower average classification value than EMG+FMG (Fig 5).

Furthermore, it was shown that during changes in position and weight, EMG and FMG together can classify the 4 hand gestures more accurately than the 2 sensing modalities alone (Fig 6). While this was not tested in a real-time control application, our results show that combining FMG with EMG yields a more accurate and less variable classification. This is likely the result of the additional information provided to the classifier by both sensing modalities. The current literature, while sparse, does not offer a consensus if the combination of the 2 sensing modalities aid in gesture classification, as some articles describe no statistical differences [34,37] while others find an improved accuracy with the combination [33,35,36]. However, these studies do not include the variation of grasped load and, to the best of our knowledge, only one study includes variations in limb position [34], both of which were found to have significant effects on gesture classification accuracy of EMG and FMG. In this study, we have found that EMG+FMG yields larger and less variable classification accuracies during changes in limb position and grasped load, illustrating that the addition of FMG to EMG aids in the classification accuracy during movement and object interaction.

### Impact of varying position and grasped load

This work underscores the importance of including variations of both position and weight into hand gesture classification models. This consideration is essential, since many applications involving gesture classification such as exoskeletons or prostheses generally require the manipulation of objects of varying weights in a variety of limb positions. We have shown that varying these 2 parameters can dramatically decrease gesture classification accuracy regardless of the employed sensing modalities (EMG, FMG, or EMG+FMG). This is an important finding, as current literature often focuses on classifying hand gestures in a neutral and unloaded position or do not include the variation in grasped load [22,45,57,63]. While our results (as shown in Fig 6) alone are not enough to completely characterize the effect of position and weight across all possible activities of daily living, it does show a stark difference between classifying hand gestures in a neutral and unloaded position compared to when the positions and weights are varied. The addition of weight alters the muscle activation as a greater weight requires more activation to hold. Combined with varying positions, these minor changes in muscle activation can greatly affect classification accuracy and result in incorrect classification. Taken together, these results point to the fact that position and weight have a significant effect on classification accuracy and should be included or considered in the development of hand gesture recognition training and testing algorithms.

### Training effect

We found that when the classifier was trained at one position and grasped load and then tested at either a different position or load, there was a significant decrease in classification accuracy and an increase in the variance of classification accuracies. As shown by Table 3 and 4, the median values from both the conditions were numerically similar, ranging from 68–75% and yielding large variance as shown by the IQR values. These results demonstrate that training and testing gesture classification algorithms in a single position leads to suboptimal classification performance if these conditions are changed. These results are expected as the variations in both position and grasped load alter muscle activations during hand gestures. Thus, if trained in either a single position or loading condition, a classifier would not be able to account for these variations and result in higher variance and lower classification accuracy, as we found. Other works have attempted to mitigate these effects by training the classifier at multiple limb positions [18,38,41,64]. While this has proven to yield better classification accuracies than training at a single position, such techniques can be time consuming and can still lead to incorrect gesture classification as testing in every position or under every possible weight may be infeasible and impractical. Other studies have used dynamic training approaches, where the subject performs a hand gesture over a set range of motion [21,39]. While these tests have yielded better classification accuracies and reduced training times, these tests omit the importance of object interactions and the imposition of grasped weight on the hand and supporting musculature, which we have shown to significantly effect gesture classification. A more robust training scheme that can adapt to these changes in positions and weights, perhaps by dynamically training the system with different weights that represent a light, medium, and heavy load, or using regression strategies could yield more accurate gesture classification. While the techniques used in this experiment highlighted the importance of limb position and grasped load, training a classifier in all the limb positions and load conditions as we did likely does not represent the most time efficient approach. Thus, a further investigation into a strategic training scheme that optimizes time demands while accommodating the varying limb positions and grasped loads is pertinent for gesture recognition devices.

Our results also point to the fact that changes in position impact classification accuracy more heavily than changes in weight. Shown in Tables 3 and 4, the IQR values from the constant weight and varying position condition were found to be numerically larger than that of the constant position and varying weight condition, ranging from 28–44% and 22–35% respectively. The higher variability caused by variations in position may be caused by additional muscle contractions that are used to support and stabilize the limb in a specific position. This ultimately could lead to incorrect hand gesture classification as the data from a single gesture in one position may be dramatically different from the same hand gesture in another position.

### Large variations in position and grasped loads

We found that large variations in limb position and grasped load effect each of the sensing modalities when they were trained in the neutral and unloaded condition (shown in Fig 9). The classification accuracy for each sensing modality was found to be statistically different when comparing the neutral condition to any of the other more extreme conditions. This was expected as training and testing at the neutral condition resulted in an accuracy close to 100% for each sensing modality (Fig 6) while training at one condition and testing in another yields variable results (Tables 3 and 4).

When comparing the other conditions, it was found that for EMG+FMG and FMG, there were no statistical differences between the loaded condition and the outstretched condition. This points to the fact that large changes in position and grasped load affect these sensing modalities to a similar degree. Interestingly, the results from Fig 9 show that EMG is more influenced by variations in limb position rather than grasped load, as there is a statistical difference between the loaded and outstretched conditions with the outstretched condition resulting in a lower classification accuracy. This is further reinforced by the fact that the combined loaded and outstretched condition is not statistically different from the outstretched condition, suggesting that the addition of load did not have as much of a significant effect on EMG classification accuracy. In fact, the results indicate that EMG is more affected by changes in position than FMG, as a significant difference was found between EMG and FMG at the outstretched condition. Changes in limb position may affect EMG more as the sensor’s position on the forearm can be altered as the limb is outstretched, causing variations in EMG recordings. As FMG relies solely on the volumetric changes of the limb, slight shifts in FMG sensor placement cause less discrepancy in FMG recordings than in EMG recordings. However, it is most important to note that the addition of these changes in position and grasped load led to a major decrease in classification accuracy for all sensing modalities for all conditions when comparing to the neutral condition. This highlights the importance of including these variations in classification models.

## Future work and conclusion

While this work examined how variations in limb position and grasped load effect EMG, FMG, and EMG+FMG, there are many opportunities to expand this experiment in future works. One of these ways is the testing of different hand gesture classification algorithms. We chose to use LDA as it is easy to implement and commonly used in muscle pattern classification [57,58]. We further wanted to establish a baseline to illustrate how variations in limb position and grasped load affect LDA, as it is low complexity but still widely used in both commercial and experimental systems. However, a more robust classifier may be able to account for changes in limb position and loading more effectively than LDA is able to. Thus, this work offers a baseline data point to begin comparing various classifiers to help identify those that best accommodate varying limb positions and grasped loads. Building off this idea, identifying features and feature sets that maximize classification performance under limb position and load effects is warranted in future work. In this work, we used low complexity and widely implemented features for EMG (the Hudgin’s Set) and FMG (MAV) and used a “stacked” method for the EMG+FMG feature, creating a performance baseline for both the position and loading effects on all 3 sensing modalities. However, there are multiple other features and feature combination techniques that can be investigated in future work to better understand those yielding the most robust classification accuracies during changes in limb position and loading, such as frequency domain features and a hierarchy-based combination strategy [37]. There may be also different feature extraction methods, such as spatial based feature extraction schemes that have been shown to be more effective when sensors are worn radially [65,66].

An important aspect of future work expanding upon this study resides in its clinical application for assistive devices such as upper limb prostheses. Advanced upper limb prostheses translate muscle activity into device movement using EMG and thus suffer from both position and loading effects. While prostheses have historically served as the primary use case for many EMG pattern recognition and FMG techniques, it is important to acknowledge that body position and grasped load are reflected as forces developed inside a prosthetic socket, a very different loading condition not captured in our work with able-bodied individuals. Nevertheless, the methodologies presented in this study can be readily adapted in future research to explore these differences seen in prostheses to allow for more effective control during variations in limb position and loading. Additionally, other assistive devices and consumer-related applications such as exoskeleton control or gesture recognition for virtual and augmented reality systems are increasingly adopting multi-sensor approaches that integrate EMG for hand gesture recognition [67]. Our data is particularly relevant to these emerging applications, where users are likely to interact with physical objects using their intact hand in various positions relative to the body. Finally, it is important to note that our work involved offline analysis. While this approach provides important insights for real-time control applications, future studies must focus on the real-time control of external devices in real-world scenarios to enhance device performance in their specific use cases.

In conclusion, we found that variations in the position and grasped weight affect gesture classification of EMG, FMG, and their combination. The combination of EMG and FMG proved to be the most robust sensing modality and more accurately classified hand gestures with less variability during changes in limb position and grasped load. However, it was shown that training in a single position and grasped load, then testing in other positions and load conditions yielded variable classifications from all sensing modalities, pointing to the fact that the current technique of training and testing in a neutral and unloaded position is not amenable to how we use our hands in daily life. Thus, as the field of gesture classification from muscle activity progresses, it is pertinent to account for these changes in position and grasped weight. Overall, this works serves as baseline for how position and loading effects alter the effectiveness of EMG, FMG and EMG+FMG gesture classification. The techniques, systems, and methodologies used in the present work can be expanded upon to investigate different classification strategies as well as be adapted to test with those living with limb difference.

## Supporting information

S1 Fig The average confusion matrix from the FMG data. (TIF)

S2 Fig The average confusion matrix from the EMG data. (TIF)

S3 Fig The average confusion matrix from the EMG+FMG data. (TIF)

S4 Fig Constant load of 0g with varying positions. The gesture classification accuracies from training and testing at various positions under a constant grasped load of 0g.
(TIF)

S5 Fig Constant load of 250g with varying positions. The gesture classification accuracies from training and testing at various positions under a constant grasped load of 250g.
(TIF)

S6 Fig Constant load of 500g with varying positions. The gesture classification accuracies from training and testing at various positions under a constant grasped load of 500g.
(TIF)

S7 Fig Constant load of 750g with varying positions. The gesture classification accuracies from training and testing at various positions under a constant grasped load of 750g.
(TIF)

S8 Fig Constant load of 1000g with varying positions. The gesture classification accuracies from training and testing at various positions under a constant grasped load of 1000g.
(TIF)

S9 Fig Constant position of position 1 with varying grasped loads. The gesture classification accuracies from training and testing at various grasped loads under a constant position of position 1.
(TIF)

S10 Fig Constant position of position 2 with varying grasped loads. The gesture classification accuracies from training and testing at various grasped loads under a constant position of position 2.
(TIF)

S11 Fig Constant position of position 3 with varying grasped loads. The gesture classification accuracies from training and testing at various grasped loads under a constant position of position 3.
(TIF)

S12 Fig Constant position of position 4 with varying grasped loads. The gesture classification accuracies from training and testing at various grasped loads under a constant position of position 4.
(TIF)

S13 Fig Constant position of position 5 with varying grasped loads. The gesture classification accuracies from training and testing at various grasped loads under a constant position of position 5.
(TIF)

S14 Fig Constant position of position 6 with varying grasped loads. The gesture classification accuracies from training and testing at various grasped loads under a constant position of position 6.
(TIF)

S15 Fig Constant position of position 7 with varying grasped loads. The gesture classification accuracies from training and testing at various grasped loads under a constant position of position 7.
(TIF)

S16 Fig Constant position of position 8 with varying grasped loads. The gesture classification accuracies from training and testing at various grasped loads under a constant position of position 8.
(TIF)
