# Supplementary figures and images for: The effects of limb position and grasped load on hand gesture classification using electromyography, force myography, and their combination
Source: PLoS One. 2025 Apr 10;20(4):e0321319. doi: 10.1371/journal.pone.0321319 (PMC11984976; doi:10.1371/journal.pone.0321319)

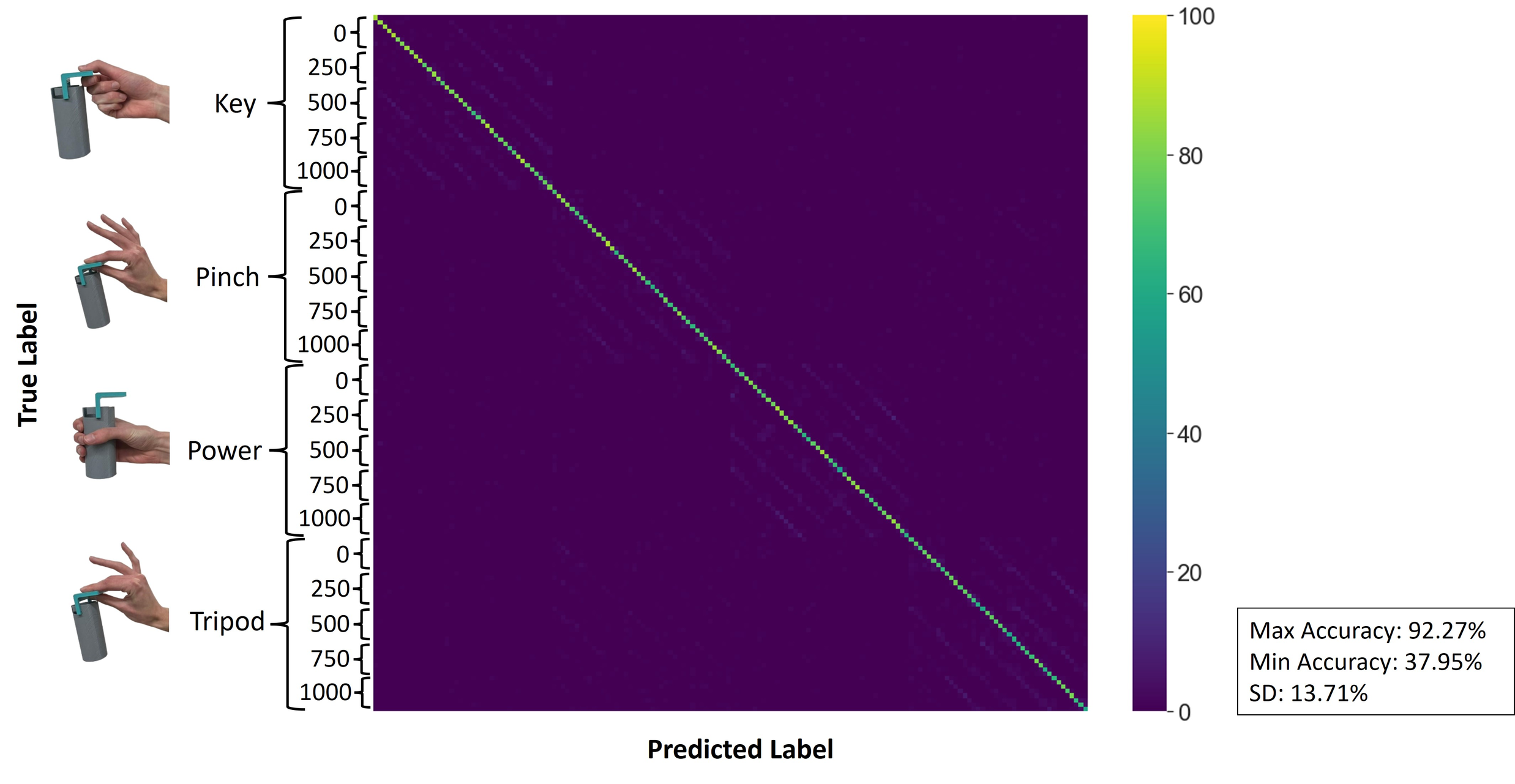

Supplement: S1 Fig — (TIF) [file pone.0321319.s001.tif]

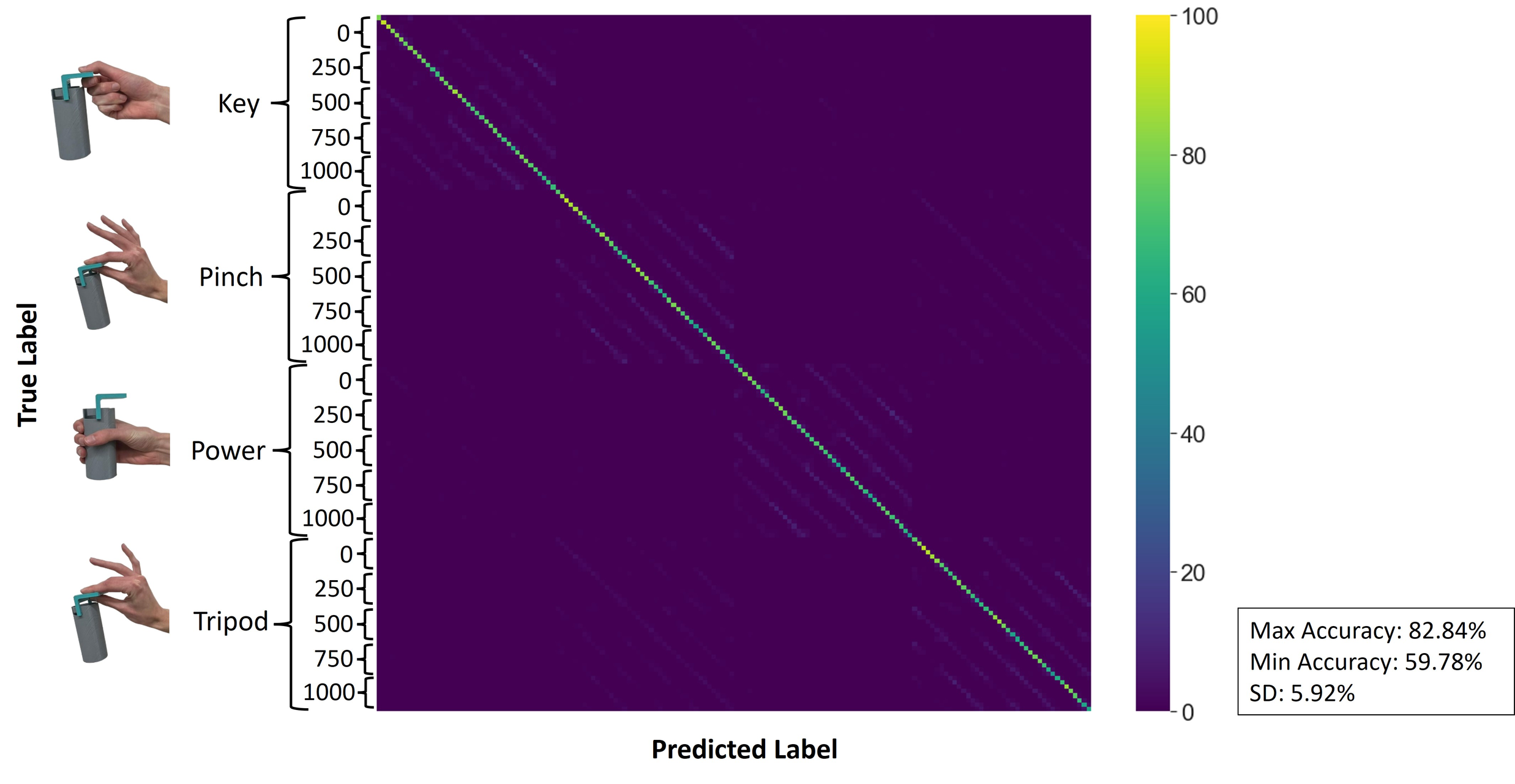

Supplement: S2 Fig — (TIF) [file pone.0321319.s002.tif]

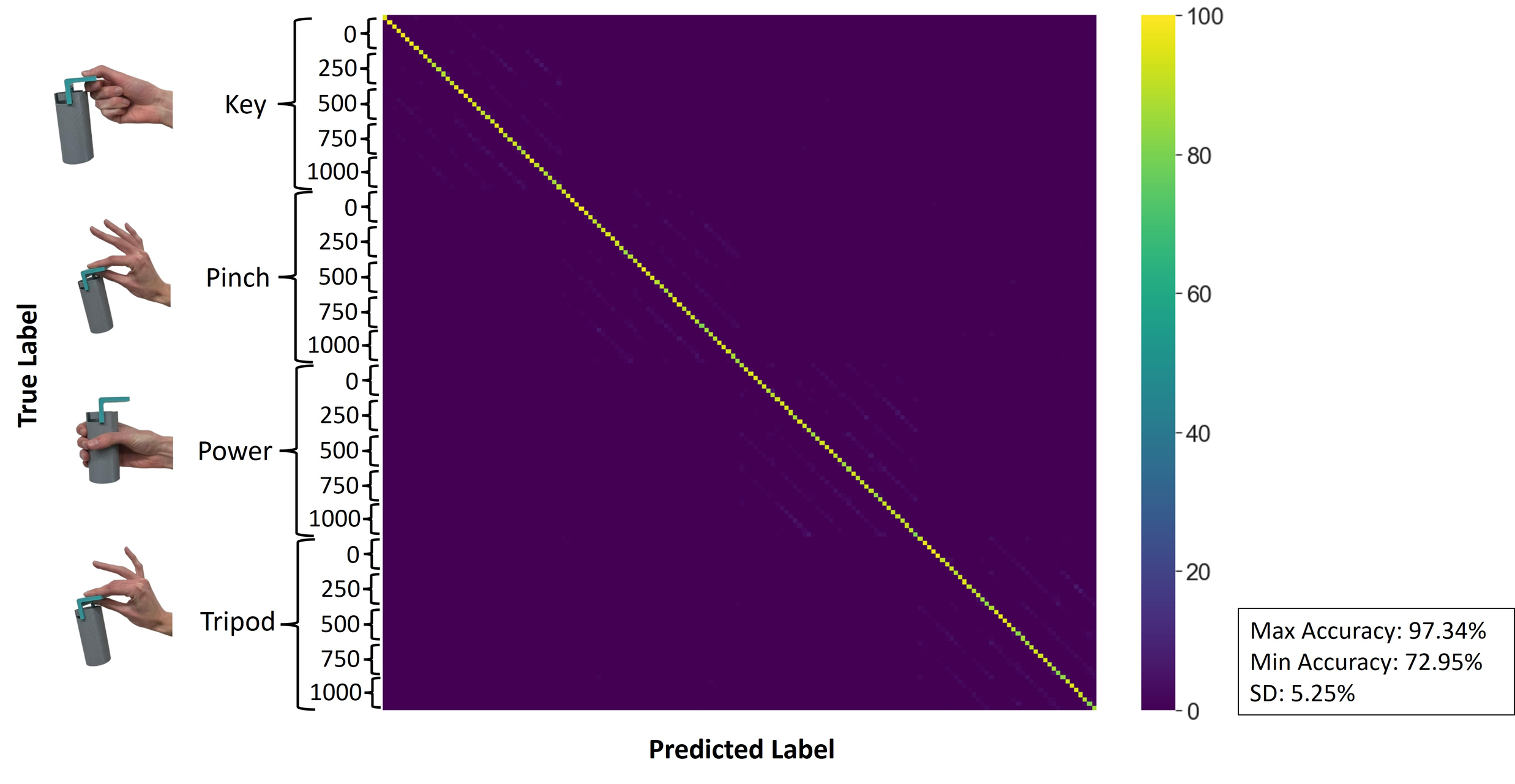

Supplement: S3 Fig — (TIF) [file pone.0321319.s003.tif]

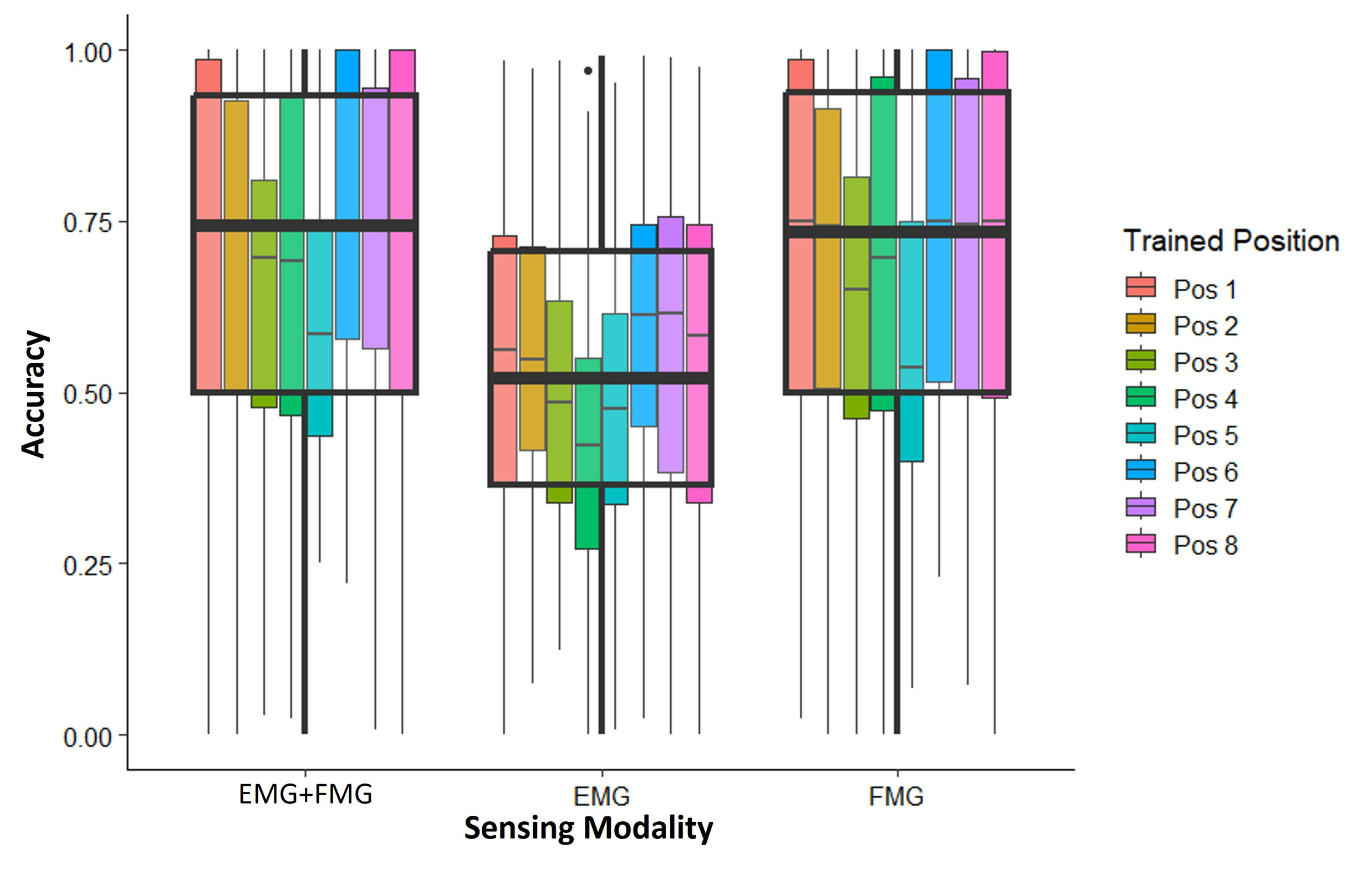

Supplement: S4 Fig — The gesture classification accuracies from training and testing at various positions under a constant grasped load of 0g. (TIF) [file pone.0321319.s004.tif]

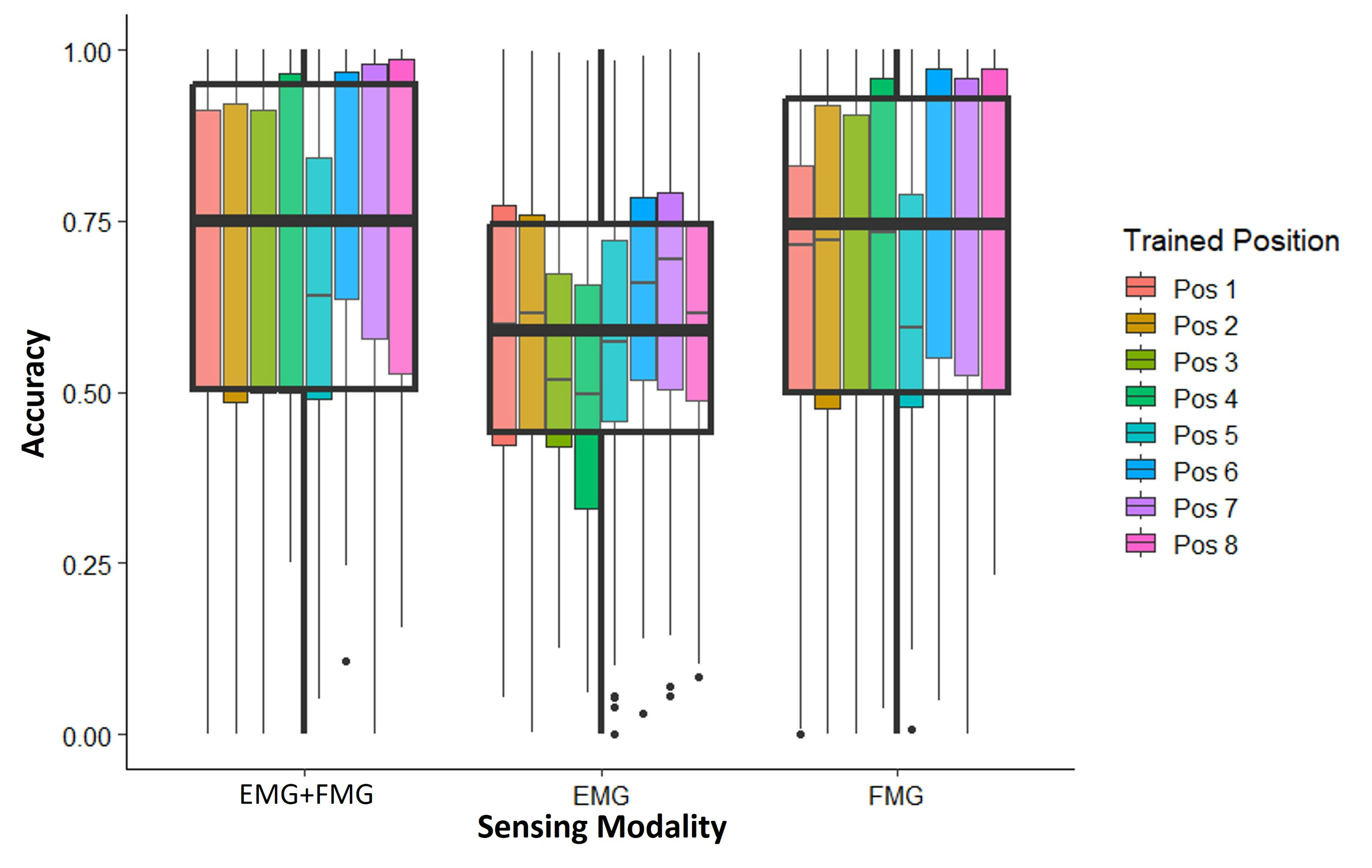

Supplement: S5 Fig — The gesture classification accuracies from training and testing at various positions under a constant grasped load of 250g. (TIF) [file pone.0321319.s005.tif]

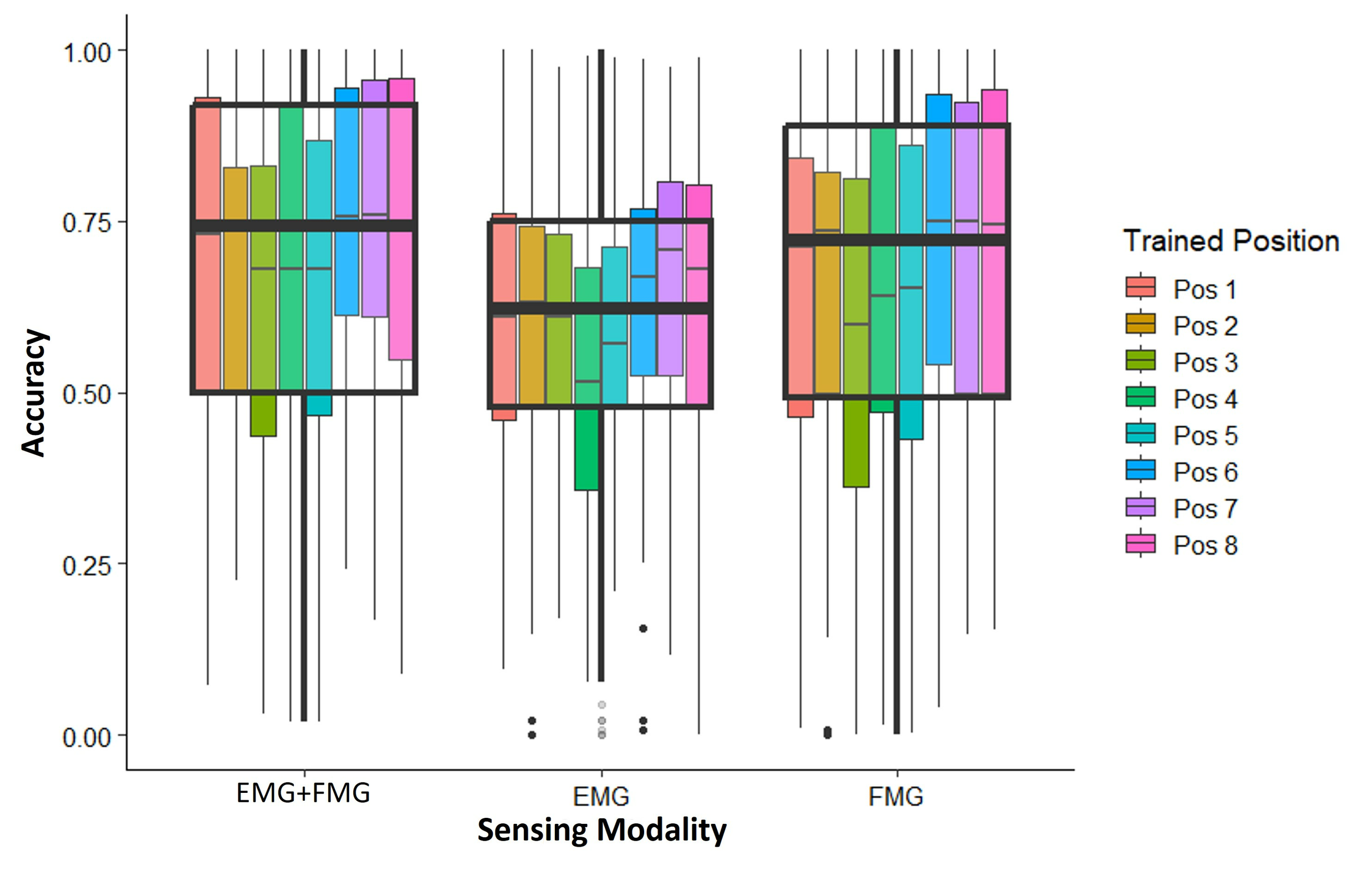

Supplement: S6 Fig — The gesture classification accuracies from training and testing at various positions under a constant grasped load of 500g. (TIF) [file pone.0321319.s006.tif]

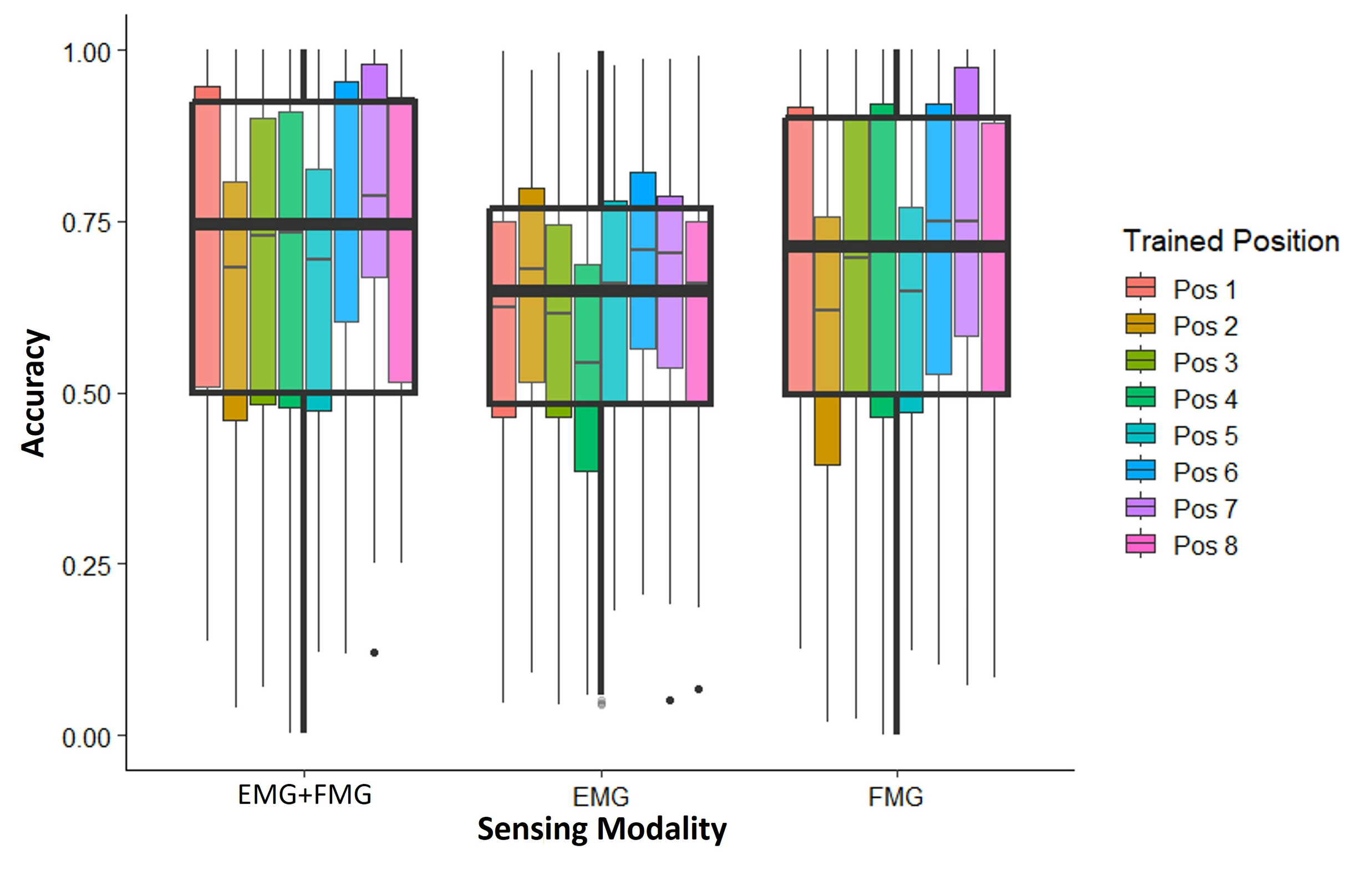

Supplement: S7 Fig — The gesture classification accuracies from training and testing at various positions under a constant grasped load of 750g. (TIF) [file pone.0321319.s007.tif]

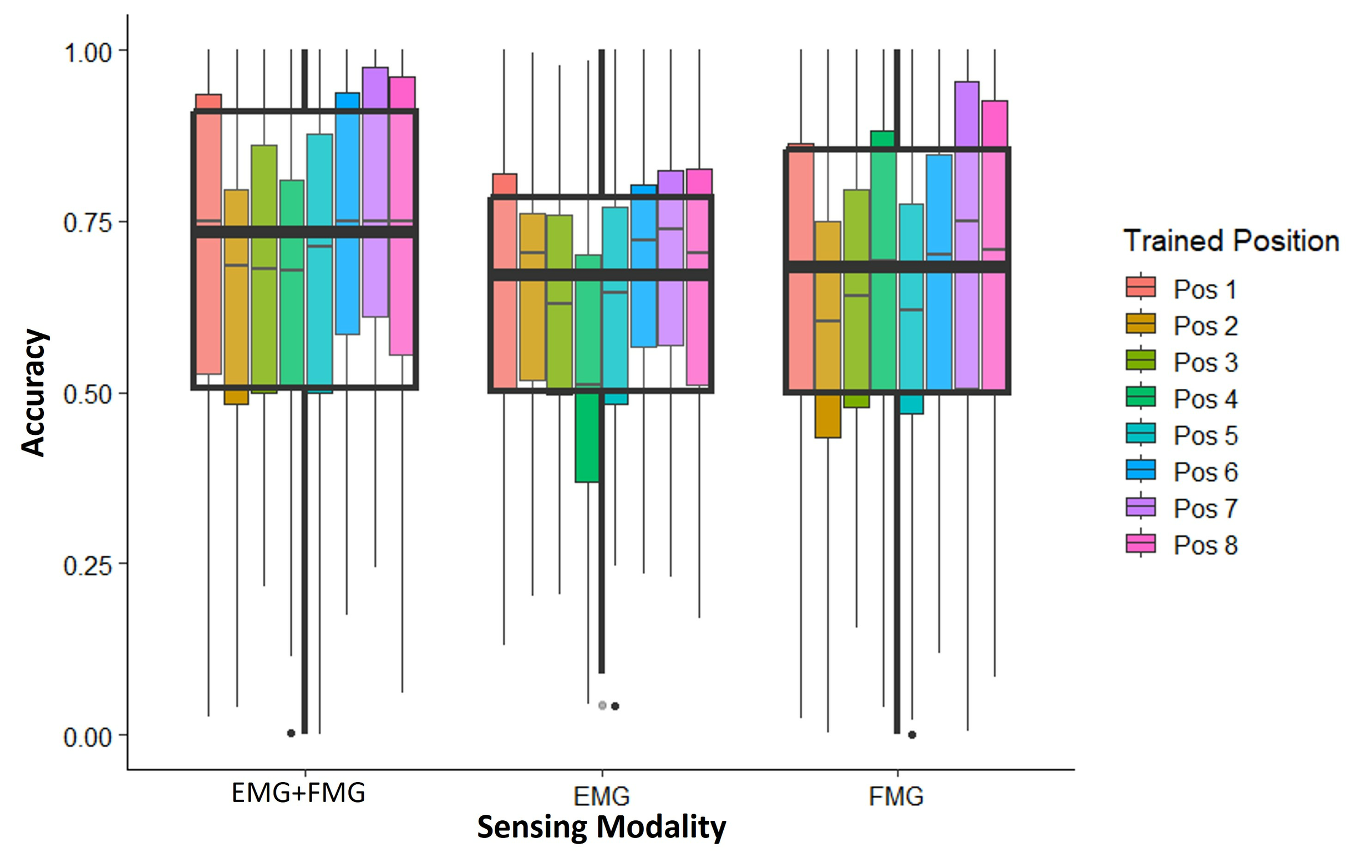

Supplement: S8 Fig — The gesture classification accuracies from training and testing at various positions under a constant grasped load of 1000g. (TIF) [file pone.0321319.s008.tif]

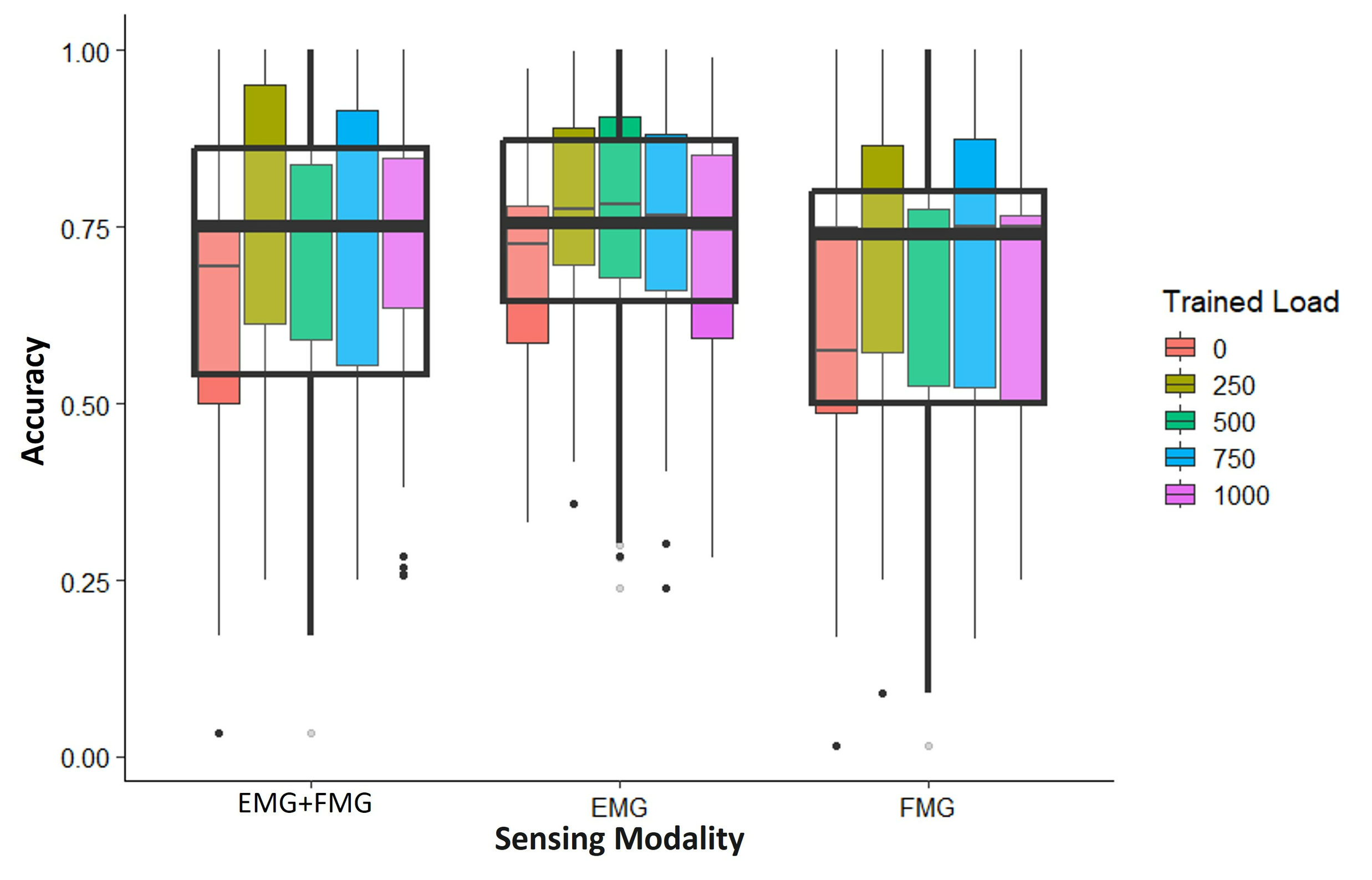

Supplement: S9 Fig — The gesture classification accuracies from training and testing at various grasped loads under a constant position of position 1. (TIF) [file pone.0321319.s009.tif]

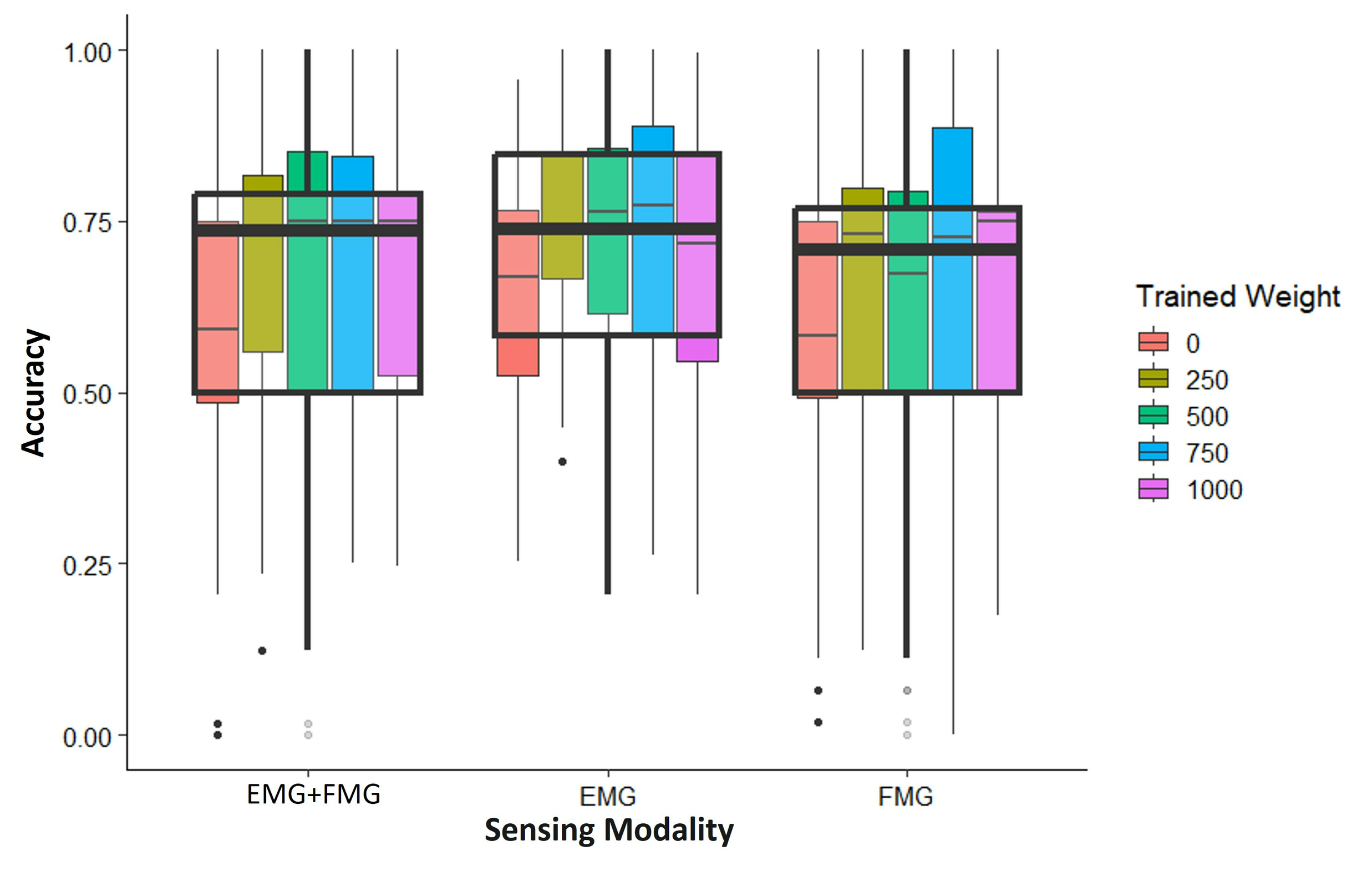

Supplement: S10 Fig — The gesture classification accuracies from training and testing at various grasped loads under a constant position of position 2. (TIF) [file pone.0321319.s010.tif]

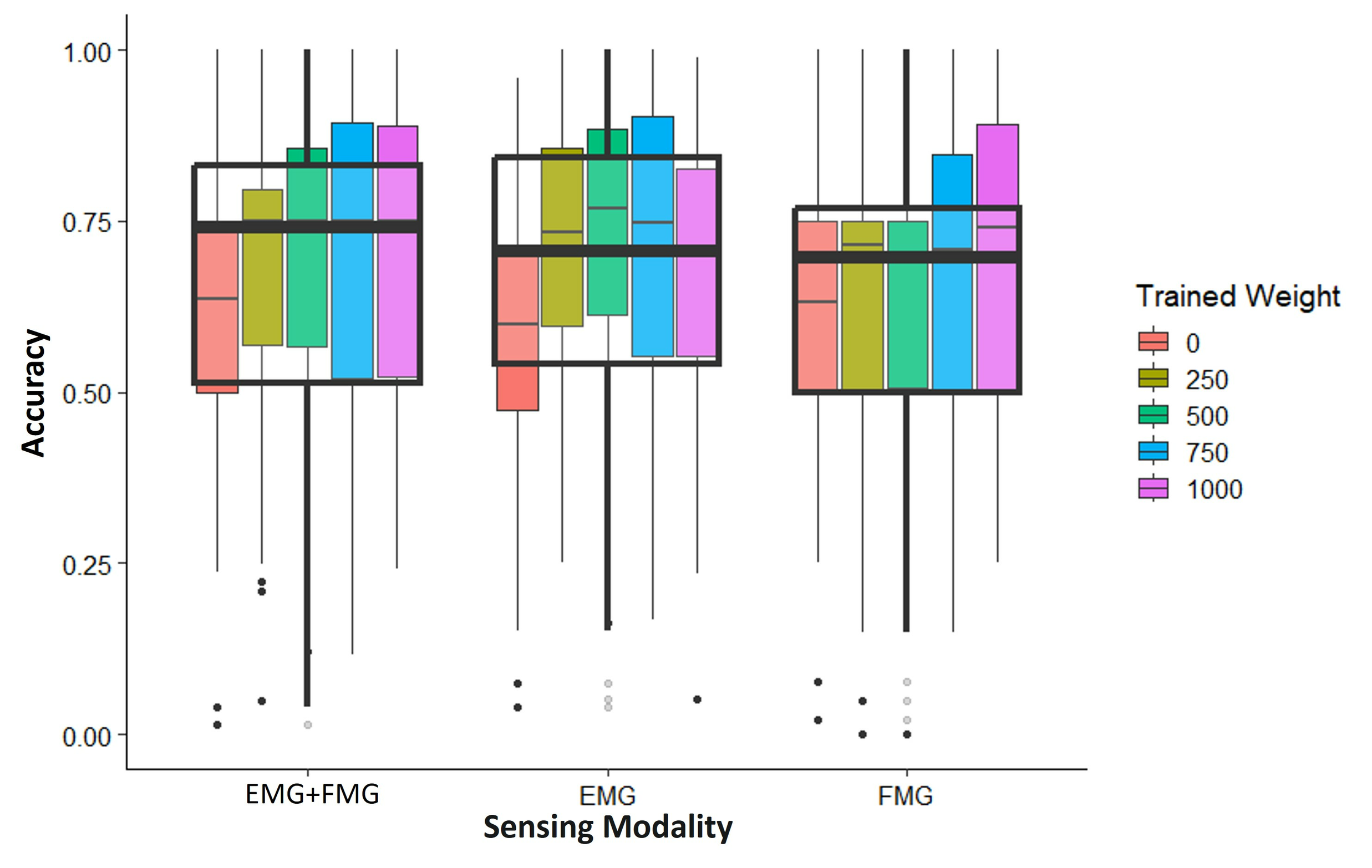

Supplement: S11 Fig — The gesture classification accuracies from training and testing at various grasped loads under a constant position of position 3. (TIF) [file pone.0321319.s011.tif]

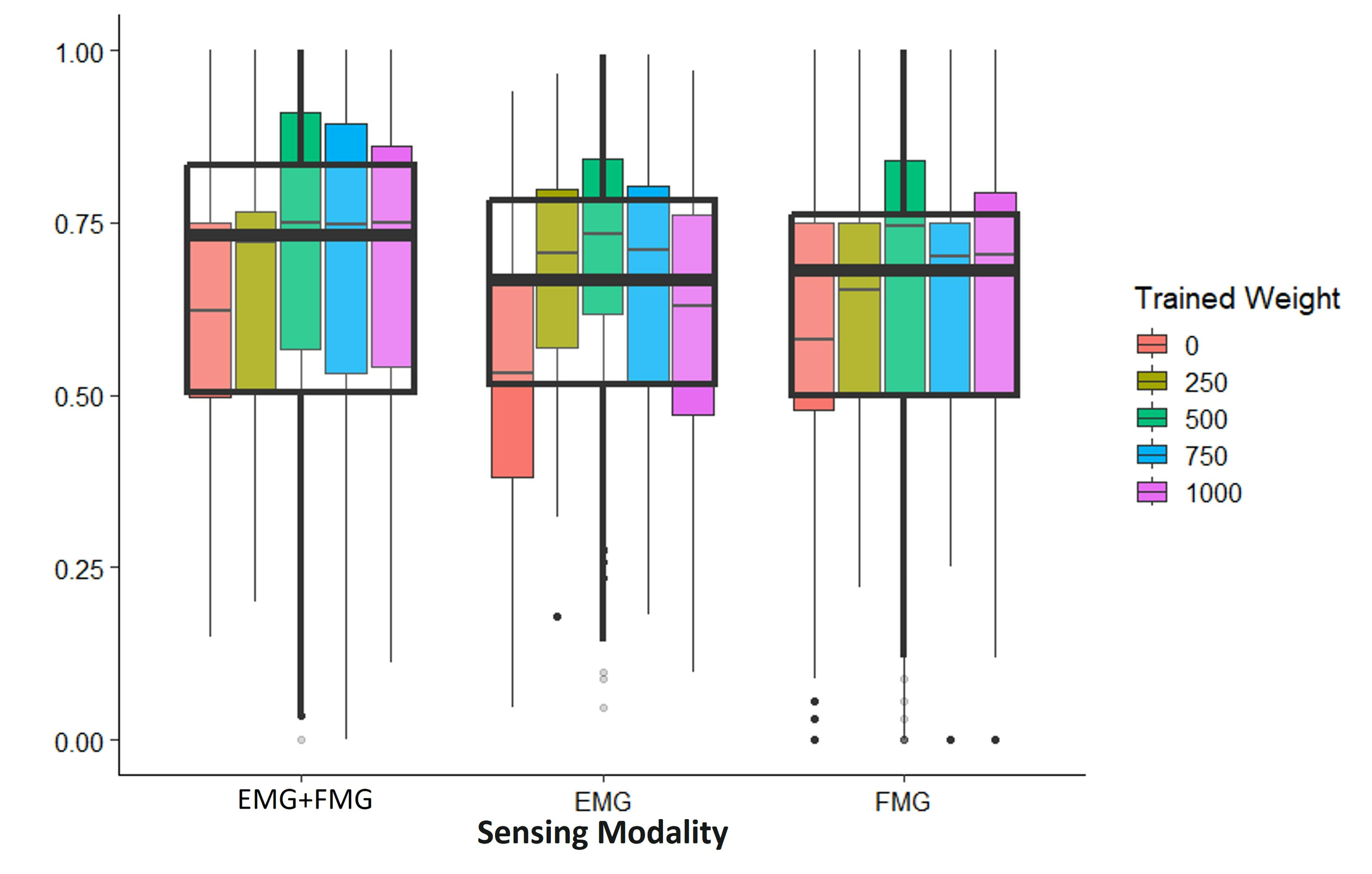

Supplement: S12 Fig — The gesture classification accuracies from training and testing at various grasped loads under a constant position of position 4. (TIF) [file pone.0321319.s012.tif]

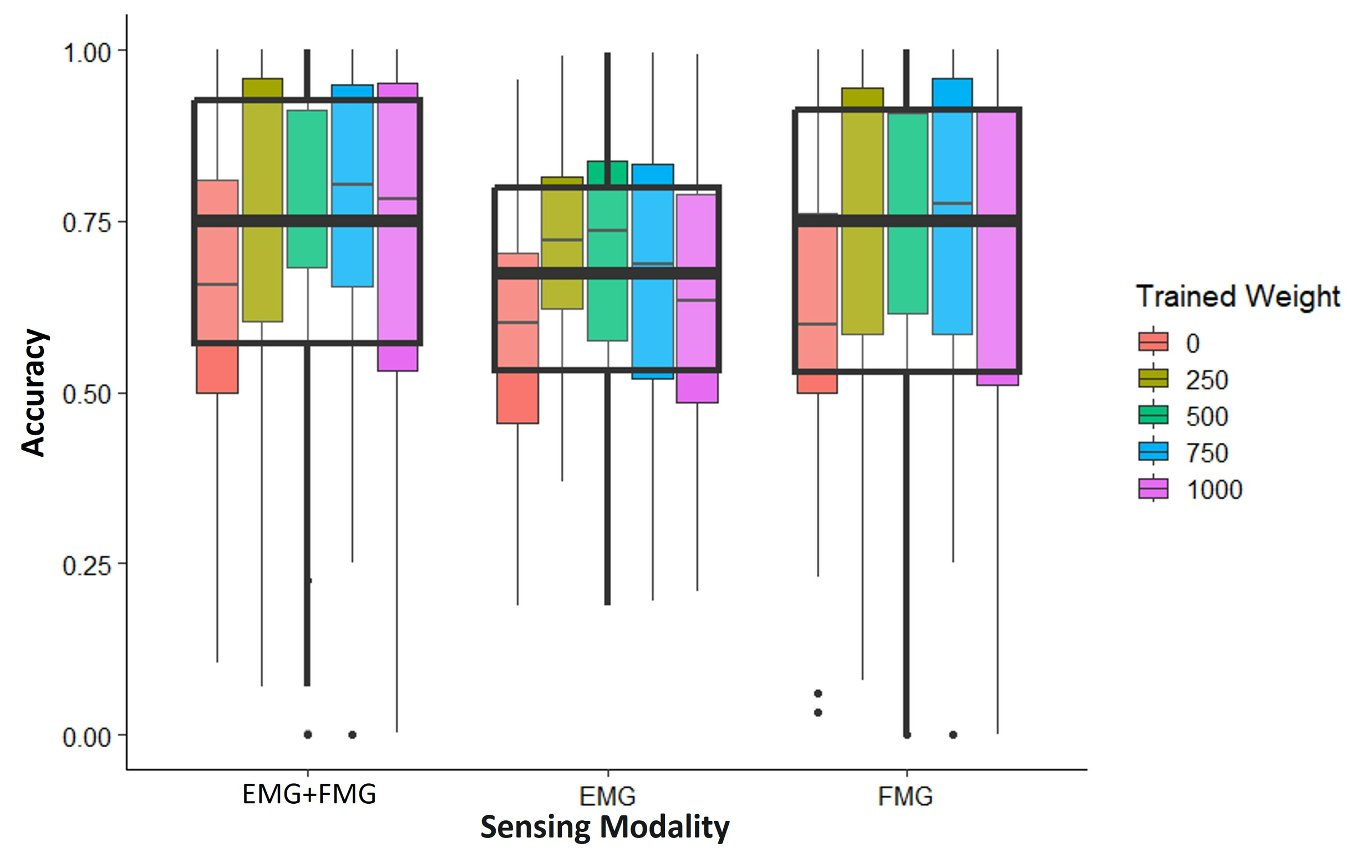

Supplement: S13 Fig — The gesture classification accuracies from training and testing at various grasped loads under a constant position of position 5. (TIF) [file pone.0321319.s013.tif]

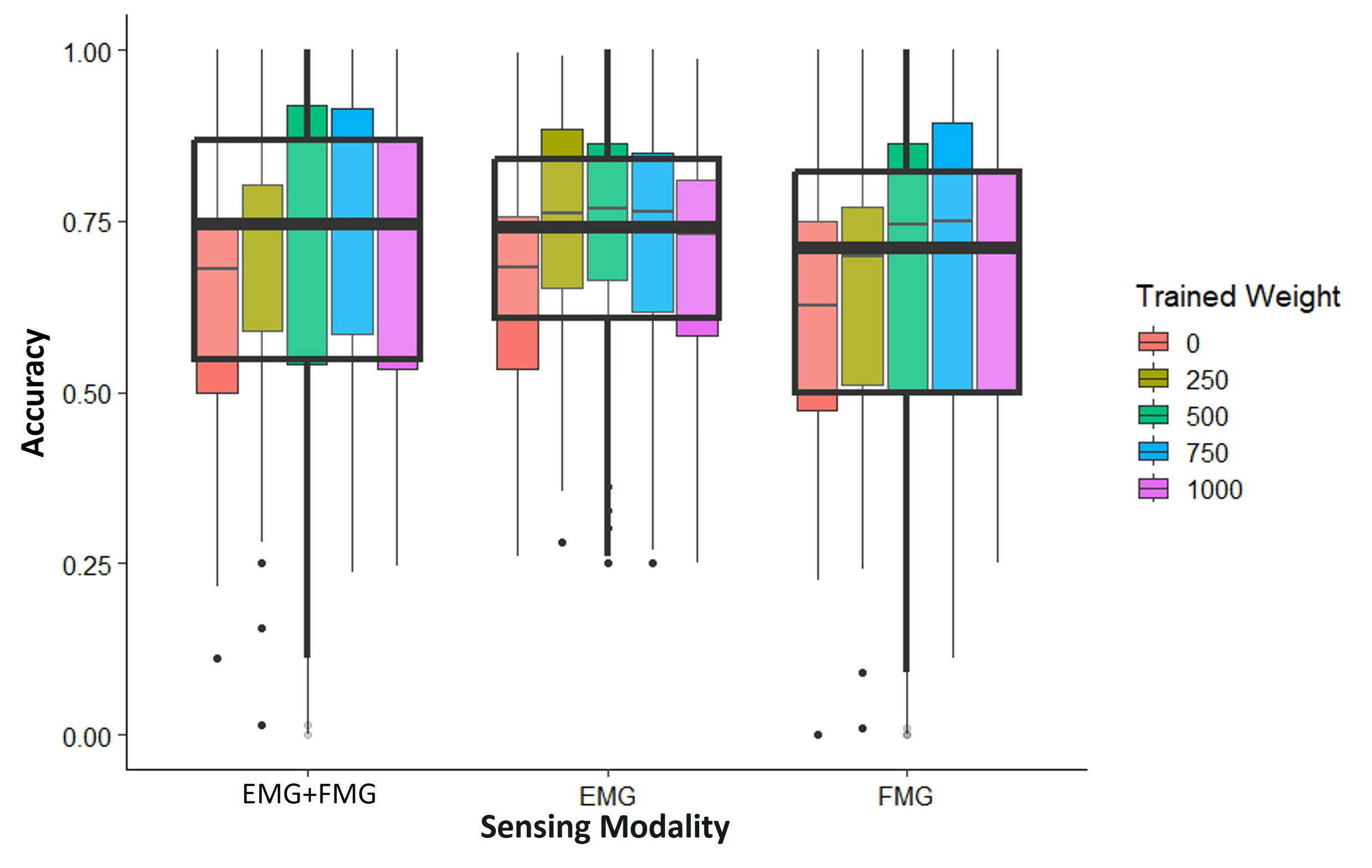

Supplement: S14 Fig — The gesture classification accuracies from training and testing at various grasped loads under a constant position of position 6. (TIF) [file pone.0321319.s014.tif]

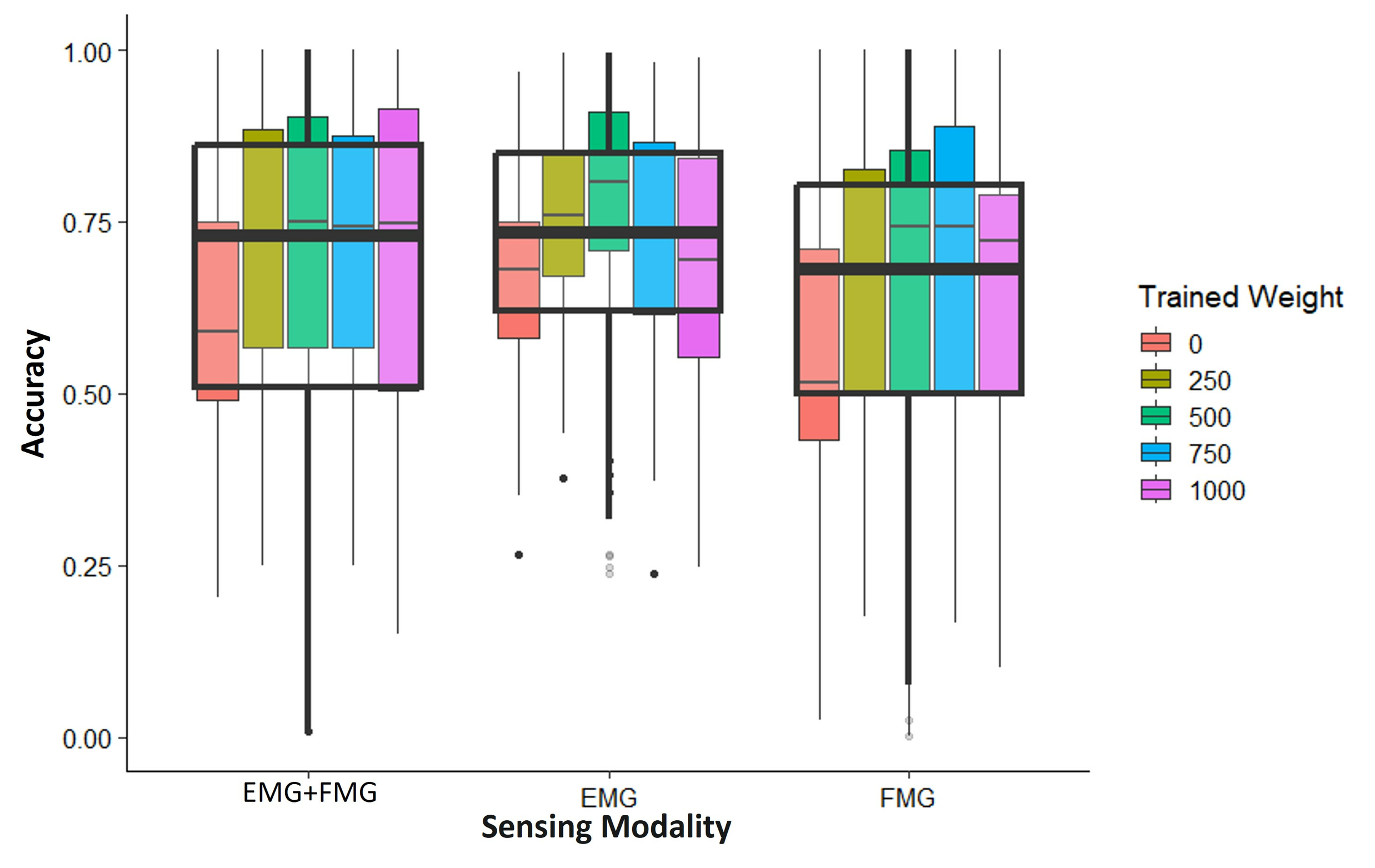

Supplement: S15 Fig — The gesture classification accuracies from training and testing at various grasped loads under a constant position of position 7. (TIF) [file pone.0321319.s015.tif]

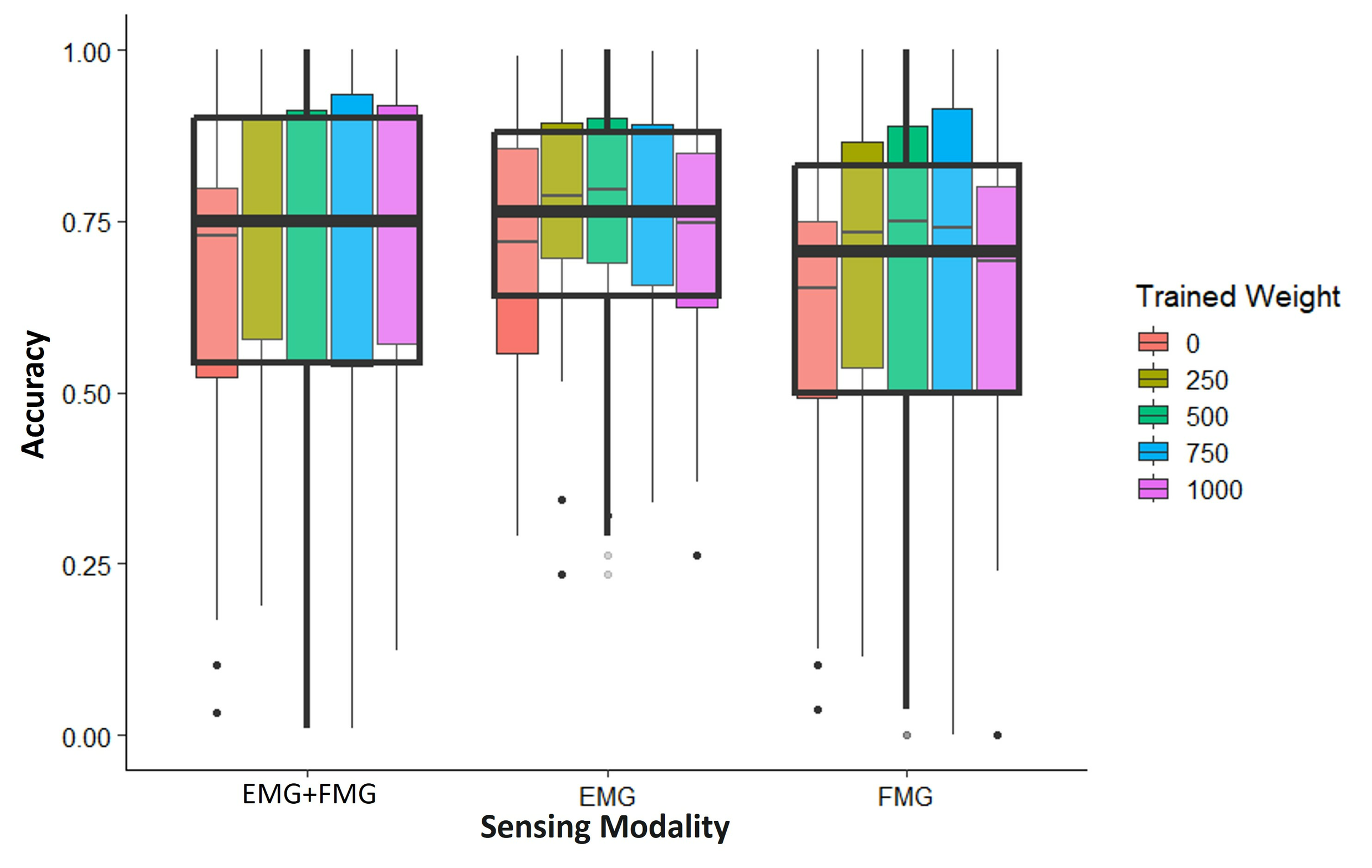

Supplement: S16 Fig — The gesture classification accuracies from training and testing at various grasped loads under a constant position of position 8. (TIF) [file pone.0321319.s016.tif]
